# A scoping review of frameworks in empirical studies and a review of dissemination frameworks

**DOI:** 10.1186/s13012-022-01225-4

**Published:** 2022-08-09

**Authors:** Ana A. Baumann, Cole Hooley, Emily Kryzer, Alexandra B. Morshed, Cassidy A. Gutner, Sara Malone, Callie Walsh-Bailey, Meagan Pilar, Brittney Sandler, Rachel G. Tabak, Stephanie Mazzucca

**Affiliations:** 1 Division of Public Health Sciences, Department of Surgery, Washington University in St. Louis, St. Louis, USA; 2 School of Social Work, Brigham Young University, Provo, USA; 3 BJC HealthCare, Community Health Improvement, St. Louis, USA; 4 Rollins School of Public Health, Emory University, Atlanta, USA; 5 ViiV Healthcare, Research Triangle Park, NC USA; 6 Department of Psychiatry, Boston University School of Medicine, Boston, MA USA; 7 Brown School of Social Work, Washington University in St. Louis, St. Louis, USA; 8 Department of Infectious Diseases, Washington University School of Medicine, Washington University in St. Louis, St. Louis, USA; 9 Bernard Becker Medical Library, School of Medicine, Washington University in St. Louis, St. Louis, USA

**Keywords:** Dissemination, Frameworks, Dissemination research

## Abstract

**Background:**

The field of dissemination and implementation (D&I) research has grown immensely in recent years. However, the field of dissemination research has not coalesced to the same degree as the field of implementation research. To advance the field of dissemination research, this review aimed to (1) identify the extent to which dissemination frameworks are used in dissemination empirical studies, (2) examine how scholars define dissemination, and (3) identify key constructs from dissemination frameworks.

**Methods:**

To achieve aims 1 and 2, we conducted a scoping review of dissemination studies published in D&I science journals. The search strategy included manuscripts published from 1985 to 2020. Articles were included if they were empirical quantitative or mixed methods studies about the dissemination of information to a professional audience. Studies were excluded if they were systematic reviews, commentaries or conceptual papers, scale-up or scale-out studies, qualitative or case studies, or descriptions of programs. To achieve aim 1, we compiled the frameworks identified in the empirical studies. To achieve aim 2, we compiled the definitions from dissemination from frameworks identified in aim 1 and from dissemination frameworks identified in a 2021 review (Tabak RG, Am J Prev Med 43:337-350, 2012). To achieve aim 3, we compile the constructs and their definitions from the frameworks.

**Findings:**

Out of 6017 studies, 89 studies were included for full-text extraction. Of these, 45 (51%) used a framework to guide the study. Across the 45 studies, 34 distinct frameworks were identified, out of which 13 (38%) defined dissemination. There is a lack of consensus on the definition of dissemination. Altogether, we identified 48 constructs, divided into 4 categories: process, determinants, strategies, and outcomes. Constructs in the frameworks are not well defined.

**Implication for D&I research:**

This study provides a critical step in the dissemination research literature by offering suggestions on how to define dissemination research and by cataloging and defining dissemination constructs. Strengthening these definitions and distinctions between D&I research could enhance scientific reproducibility and advance the field of dissemination research.

**Supplementary Information:**

The online version contains supplementary material available at 10.1186/s13012-022-01225-4.

Contributions to the literature

- The field of dissemination research has not coalesced to the same degree as the field of implementation research. Clearly defining dissemination and identifying dissemination constructs will help enhance dissemination research.
- In a review of 34 frameworks, we found a lack of consensus in the definition of dissemination and 48 constructs identified in the frameworks.
- We provide a suggested definition of dissemination and a catalog of the constructs to advance the field of dissemination research.

## Background

The field of dissemination and implementation (D&I) research has grown extensively in the past years. While scholars from the field of *implementation research* have made substantial advances, the field of *dissemination research* has not coalesced to the same degree, limiting the ability to conduct rigorous, reproducible dissemination research. Dissemination research has broadly focused on examining how evidence-based information gets packaged into practices, policies, and programs. This information delivery is often targeted at providers in public health and clinical settings and policymakers to improve public health decision-making. Here, we use *provider* to refer to a person or group that provides something—in this case, information. The chasm between how evidence-based information is disseminated and how this information is used by providers and policymakers is well-documented [1] and further evidenced by the ongoing COVID-19 pandemic [2, 3].

The definition of dissemination research has been modified over the years and is not consistent across various sources. Dissemination research could be advanced by further development of existing conceptual and theoretical work. In a previous review [4], nine D&I science frameworks were categorized as “dissemination only” frameworks (i.e., the explicit focus of the framework was on the spread of information about evidence-based interventions to a target audience) [4]. Frameworks are important because they provide a systematic way to develop, plan, manage and evaluate a study [5, 6]. The extent to which dissemination scholars are using frameworks to inform their studies, and which frameworks are used, is unclear.

Building on previous compilations of dissemination frameworks [7], this paper intends to advance the knowledge of dissemination research by examining dissemination frameworks reported in the empirical literature, cataloging the constructs across different frameworks, and providing definitions for these constructs. A scoping review is ideal at this stage of the dissemination research literature because it helps map the existing frameworks from a body of emerging literature and identifies gaps in the field [8].

Specifically, this study has three aims: (1) to conduct a scoping review of the empirical dissemination literature and identify the dissemination frameworks informing those studies, (2) to examine how scholars define dissemination, and (3) to catalog and define the constructs from the dissemination frameworks identified in aim 1 and the frameworks categorized as dissemination only by Tabak et al. [4]

## Methods

The methods section is divided into the three aims of this study. First, we report the methods for our scoping review to identify the frameworks used in empirical dissemination studies. Second, we report on how we identified the definitions of dissemination. Third, we report the methods for abstracting the dissemination constructs from the frameworks identified in the empirical literature (aim 1) and from the frameworks categorized as “dissemination only” by Tabak et al. [4] Tabak et al. [4] categorized models “on a continuum from dissemination to implementation” and acknowledge that “these divisions are intended to assist the reader in model selection, rather than to provide actual classifications for models.” For the current review, we selected only those categorized as dissemination-only because we aimed to examine whether there were any distinct components between the dissemination and implementation frameworks by coding the dissemination-only frameworks.

### Scoping review of the literature

We conducted a scoping review to identify dissemination frameworks used in the empirical dissemination literature. A scoping review is appropriate as the goal of this work is to map the current state of the literature, not to evaluate evidence or provide specific recommendations as is the case with a systematic review [8]. We followed the method developed by Arksey and O’Malley [9] and later modified by Levac and colleagues [10]. In doing so, we first identified the research questions (i.e., “Which dissemination frameworks are used in the literature?” and “How are the dissemination constructs defined?”), identified relevant studies (see below), and charted the data to present a summary of our results.

We iteratively created a search strategy in Scopus with terms relevant to dissemination. We ran the search in 2017 and again in December 2020, using the following terms: TITLE-ABS-KEY (dissem* OR (knowledge AND trans*) OR diffuse* OR spread*) in the 20 most relevant journals for the D&I science field, identified by Norton et al. [11] We ran an identical search at a second time point due to several logistical reasons. This review was an unfunded project conducted by faculty and students who experienced numerous significant life transitions during the project period. We anticipated the original search would be out of date by the time of submission for publication, thus wanted to provide the most up-to-date literature feasible given the time needed to complete the review steps. This approach is appropriate for systematic and scoping reviews [12]

We included studies if they were (a) quantitative or mixed methods empirical studies, (b) if they were about the dissemination of information (e.g., guidelines) to targeted professional audiences, and (c) published since 1985. Articles were excluded if they were (a) systematic reviews, commentaries, or other non-empirical articles; (b) qualitative studies; (c) scale-up studies (i.e., expanding a program into additional delivery settings); (d) case studies or description of programs; and/or (e) dissemination of information to lay consumer audiences or the general public. Some of the exclusion criteria, specifically around distinguishing studies that were dissemination studies from scale-up or health communication studies, were refined as we reviewed the paper abstracts. In the “Definition of dissemination section, we explain our rationale and process to distinguish these types of studies.

The screening procedures were piloted among all coders with a random sample of articles. AB, SaM, CH, CG, EK, and CWB screened titles for inclusion/exclusion independently, then met to ensure a shared understanding of the criteria and to generate consensus. The same coders then reviewed titles based on the above inclusion/exclusion criteria. Any unclear records were retained for abstract review. Consistent with the previously utilized methodology, the abstract review was conducted sequentially to the title review [13, 14]. This approach can improve efficiency while maintaining accuracy [15]. In this round of review, abstracts were single-screened for inclusion/exclusion. Then, 26% of the articles were independently co-screened by pairs of coders; coding pairs met to generate consensus on disagreements.

Articles that passed to full-text review were independently screened by two coders (AB, CH, EK, and CWB). Coders met to reach a consensus and a third reviewer was consulted if the pair could not reach an agreement. From included records, coders extracted bibliometric information about the article (authors, journal, and year of publication) and the name of the framework used in the study (if a framework was used). Coders met regularly to discuss any discrepancies in coding and to generate consensus; final decisions were made by a third reviewer if necessary.

### Review of definitions of dissemination

First, we compiled the list of frameworks identified in the empirical studies. Because some frameworks categorized as dissemination-only by the review of frameworks in Tabak et al. [4] were not present in our sample, we added those to our list of frameworks to review. From the articles describing these frameworks, we extracted dissemination definitions, constructs, and construct definitions. AB, SM, AM, and MP independently abstracted and compared the constructs’ definitions.

### Review of dissemination constructs

Once constructs were identified, the frequency of the constructs was counted, and definitions were abstracted. We then organized the constructs into four categories: dissemination processes, determinants, strategies, and outcomes. These categories were organized based on themes by AB and reviewed by all authors. We presented different versions of these categories to groups of stakeholders along our process, including posters at the 2019 and 2021 Conferences on the Science of Dissemination and Implementation in Health, the Washington University Network for Dissemination and Implementation Researchers (WUNDIR), and our network of D&I research peers. During these presentations and among our internal authorship group, we received feedback that the categorization of the constructs was helpful.

We defined the constructs in the *dissemination process* as constructs that relate to processes, stages, or events by which the dissemination process happens. The *dissemination determinants* construct encompasses constructs that may facilitate or obstruct the dissemination process (i.e., barriers or facilitators). The *dissemination strategies* constructs are those that describe the approaches or actions of a dissemination process. Finally, *dissemination outcomes* are the identified dissemination outcomes in the frameworks (distinct from health service, clinical, or population health outcomes). These categories are subjective and defined by the study team. The tables in Additional file 1 include our suggested labels and definitions for the constructs within these four categories, the definitions as provided by the articles describing the frameworks, and the total frequency of each construct from the frameworks reviewed.

## Results

### Scoping review of the literature

**Fig. 1:**
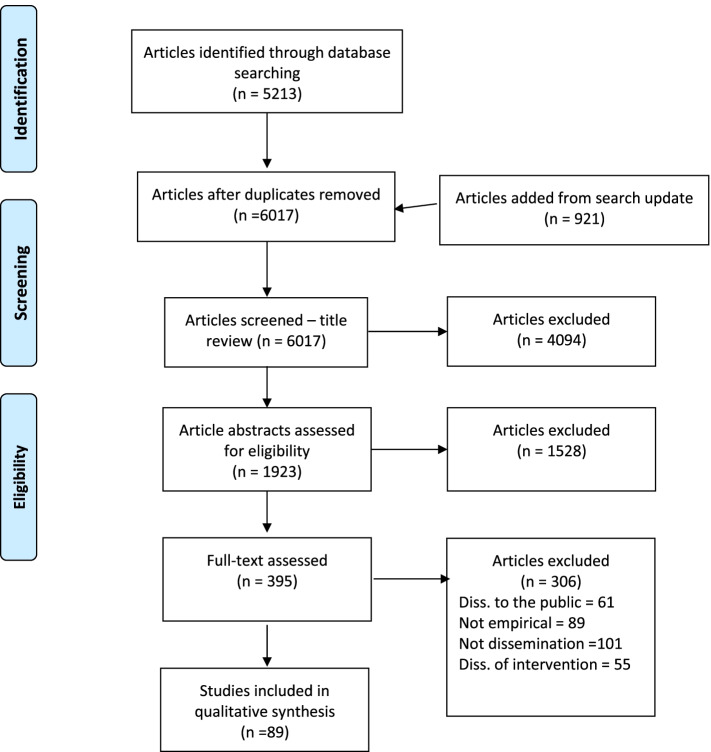
PRISMA chart

The PRISMA Extension for Scoping Reviews (PRISMA-ScR) flowchart is shown in Fig. 1. The combined searches yielded 6017 unique articles. Of those, 5622 were excluded during the title and abstract screening. Of the 395 full-text articles, we retained 89 in our final sample.

Papers were excluded during the full-text review for several reasons. Many papers (*n* = 101, 33%) were excluded because they did not meet the coding definitions for dissemination studies. For example, some studies were focused on larger quality improvement initiatives without a clear dissemination component while other studies reported disseminating findings tangentially. Many (*n* = 61; 20%) were excluded because they reported a study testing approaches to spread information to the general public or lay audiences instead of to a group of professionals (e.g., disseminating information about HIV perinatal transmission to mothers, not healthcare providers.) Several articles (*n* = 55, 18%) were related to the scale up of interventions and not the dissemination of information.

### Frameworks identified

Table 1 shows the frameworks used in the included studies. We identified a total of 27 unique frameworks in the empirical studies. Out of the 27 frameworks identified, only three overlapped with the 11 frameworks cataloged as “dissemination only” in Tabak et al. [4] review. Two frameworks identified in the empirical studies were cataloged by Tabak et al [4] as “D = I,” one was cataloged as “D > I,” and one as “I only.” Additional file 1: Table S1 shows all the frameworks, with frameworks 1–11 being “D only” from Tabak et al. [4], and frameworks 12–34 are the ones identified in our empirical sample. Rogers’ diffusion of innovation [16] was used most frequently (in 10 studies), followed by the Knowledge to Action Framework (in 4 studies) [17] and RE-AIM (in 3 studies) [18]. Dobbins’ Framework for the Dissemination and Utilization of Research for Health-Care Policy and Practice [19], the Interactive Systems Framework and Network Theory [20], and Kingdon’s Multiple Streams Framework [21] were each used by two studies. Thirty studies (33%) did not explicitly describe a dissemination framework that informed their work.

**Table 1:** Frequency of frameworks used in the dissemination studies from our sample (*N* = 89)

Frameworks	Number
Diffusion of Innovations^a^	10
Knowledge to Action Framework	4
RE-AIM^b^	3
Dobbins’ Framework for the Dissemination and Utilization of Research for Health-Care Policy and Practice^c^	2
Kingdon’s Multiple Streams Framework^a^	2
Interactive Systems Framework^b^	2
Network Theory	2
Affective Reactions Model	1
COM-B Model	1
Conceptual Framework for Research Knowledge Transfer and Utilization^a^	1
Edquist’s Model of Process and Product Innovation	1
Experimental Social Innovation and Dissemination	1
Information Processing Model	1
Institutional Theory	1
Interaction Model of Knowledge Translation	1
Kumagai’s Conceptual Framework for the Use of Illness Narratives in Medical Education	1
Medical Research Councils’ Theory of Change	1
Miller’s Framework for Clinical Assessment	1
Physical Activity Policy Research Framework	1
Promoting Action on Research Implementation in Health Services (PARIHS)^d^	1
SPIRIT Action Framework	1
Systematic Review of Dissemination Planning Frameworks and Strategies	1
Technology Acceptance Model	1
Thacker’s Framework for Environmental Health Surveillance	1
Theory of Middle Managers’ Roles in Healthcare EBP Implementation	1
Theory of Planned Behavior	1
Weingarden’s Stages of Implementation Model	1
No specified theory, model, or framework	44

^a^Identified as D only framework in Tabak et al.

^b^Identified as D = I in Tabak et al.

^c^Identified as D > I in Tabak et al.

^d^Identified as I only at Tabak et al. Frameworks with no note were not identified in Tabak et al.

### Definition of dissemination

Table 2 shows the definition of dissemination from the frameworks. Out of the 38 frameworks, only 12 (32%) defined dissemination. There is wide variability in the depth of the definitions, with some authors defining dissemination as a process “transferring research to the users,” [24] and others defining it as both a process and an outcome [19, 23]. The definitions of dissemination varied among the 13 frameworks that defined dissemination; however, some shared characteristics were identified. In nine of the 13 frameworks, the definition of dissemination included language about the movement or spread of something, whether an idea, innovation, program, or research finding [16, 23–28, 31, 32]. Seven of the frameworks described dissemination as active, intentional, or planned by those leading a dissemination effort [7, 16, 23, 25–27, 32]. Five frameworks specified some type of outcome as a result of dissemination (e.g., the adoption of an innovation or awareness of research results) [7, 19, 23, 27, 29, 30]. Three of the frameworks’ definitions included the role of influential determinants of dissemination [19, 27, 29, 30]. Only two frameworks highlighted dissemination as a process [23, 25].

**Table 2:** Definition of dissemination across the dissemination frameworks

Frameworks	Definition of dissemination
Framework 1: Rogers’ Diffusion of Innovation [16]	“Dissemination is the diffusion that is directed and managed Diffusion is the planned and spontaneous spread of new ideas.” (pg. 6)
Framework 2: RAND model of persuasive communication and diffusion of medical innovation [22]	“Dissemination and acceptance of medical technology assessments can be understood within the context of theories of diffusion of innovation and of persuasive communication.” (p.314)
Framework 3: Effective dissemination strategies [23]	“Dissemination is therefore seen as a process that aims to ensure that key messages are conveyed to specified groups via a wide range of methods such that it results in some reaction, some impact or implementation.” (p.70)
Framework 4: Model for locally based research transfer development [24]	“Transferring research to the users” (pg. 1008)
Framework 8: Conceptualizing dissemination research and activity: Canadian heart health initiative [25]	“Whereas some diffusion processes can be characterized as passive or natural processes, others involve directed diffusion, or dissemination; that is, an active, deliberate, planned process to spread an innovation.” (pg. 271)
Framework 9: Policy framework for increasing diffusion of evidence-based physical activity interventions [26]	“Dissemination is the set of planned, systematic efforts designed to make a program or innovation more widely available; diffusion is the direct or indirect outcomes of those efforts.” (pg. S35)
Framework 10: Blueprint for dissemination [27]	“The various factors that influence the spread of innovation are on a continuum between pure diffusion (in which spread occurs spontaneously through decentralized and informal efforts) and active dissemination (in which spread occurs purposefully through centralized and formal efforts). This report focuses on active dissemination, that is, planned efforts to persuade targeted groups to adopt an innovation.” (pg. 2)
Framework 12: Knowledge to Action Framework [ 28]	“The spreading of knowledge or research, such as is done in scientific journals and at scientific conferences.”
Framework 14: Dobbins’ Framework for the Dissemination and Utilization of Research for Health-Care Policy and Practice [19]	“Dissemination research, defined as the study of the processes and variables that determine and/or influence the adoption of knowledge, interventions or practice by various stakeholders … ”
Framework 22: Interaction Model of Research Use: [29, 30]	“ … Dissemination is deemed to occur when a potential user becomes aware of the research results. This model explains knowledge utilization with the recourse to two determinants: the types of research results and the dissemination effort.” (22a)
Framework 32: Theory of Middle Managers’ Roles in Healthcare EBP Implementation [31]	“Diffusing information: Middle managers disseminate facts, giving employees necessary information about innovation implementation.” (pg. 5)

### Definition of dissemination constructs

Below, we describe the results presented in Tables 3, 4, 5, and 6 with constructs grouped by dissemination process, determinants, strategies, and outcomes. The definitions proposed for the constructs were based on a thematic review of the definitions provided in the articles, which can be found in the Additional file 1: Tables S2-S5.

**Table 3:** Dissemination process constructs, suggested definition, frequency of construct across frameworks, and other names in the literature

Dissemination process	Suggested definition	Frequency of constructs	Other names in the literature
Knowledge inquiry	Inquiry about the knowledge gap: examine what is known, who to approach, how to approach, why to approach stakeholders to achieve the change in the context.	6	Knowledge inquiry
Knowledge synthesis	Synthesizing the information to help make sense of the relevant knowledge.	5	Knowledge synthesis
Communication	The process of creating and sharing information with others. To distinguish communication from interaction, we conceptualize communication as a one-way communication from researchers to the audience.	3	Communication
Interaction	The process where there is an interaction and exchange of information between researchers and the audience.	7	Interaction
Persuading	The process of proactively communicating the information, including adding components such as quality gap and value added to the information.	2	Persuading
Activation	When the audience starts to act based on the information received.	2	Activation
Research transfer	When the information received becomes independent of the agent and is transferred to the audience; that is, the receiver interprets the message, draws a connection between the message and previous knowledge, and attaches meaning to the message to adopt it or reject it.	5	Research transfer, the innovation-decision process, diffusion

**Table 4:** Dissemination determinants constructs, suggested definition, frequency of construct across frameworks, and other names in the literature

Determinant constructs	Suggested definition	Total frequency	Other names in the literature
Source of knowledge	The individual or unit that delivers the information.	10	Type of source, originator of the message or knowledge, decision-makers, intervention agents, interventionists
Medium of communication	The means (form) by which the information is shared.	9	Type of communication channel, medium of communication, knowledge broker
Content of communication	The content of the message sharing the information.	14	Type of message content, format, information, innovation
Audience	Person or group receiving the information.	10	Type of user/audience/recipient/decision-maker
Type of innovation	The type or characteristics and value added of the innovation that is being communicated.	3	Type of innovation
Complexity of the innovation	The degree of complexity of an innovation being communicated.	4	Complexity of the innovation
Timing of information	The speed and distance of the spread of the information.	5	Timing of information spread
Urgency of the innovation	The urgency related to the innovation; how immediate is the need to disseminate the information about this innovation.	6	Urgency of the innovation
Triability of an innovation	The degree to which an innovation can be implemented on a limited basis.	3	Trialability of the innovation
Observability of the results	The degree to which the uptake of the innovation yields observable results.	2	Observability of the innovation’s results
Salience of the innovation	The relevance of the innovation to the audience.	14	Salience, evidence of need and demand, relative advantage of the innovation
Users’ perceived attitude towards the innovation	A more general concept than the salience of innovation, related to the audience’s perception of the process of innovation development (research) and the receptivity of the innovation.	14	Users’ attitude towards research and the innovation
Compatibility of the innovation with the setting	The degree to which an innovation is consistent with the context.	4	Compatibility of the innovation with the setting
Context	Settings in which communications are received and potential adoption occurs.	13	Context
Interpersonal networks	Large umbrella term that includes the relationship between the audience members, its structure, and its quality.	12	Influence, quality of relationships, interpersonal channels, trustworthiness, linkage mechanisms
Opinion leaders and change agents	Opinion leadership is the degree to which an individual is able to influence other individual’s attitudes. Change agent is an individual who influences a client’s innovation decisions in a certain direction and speed.	8	Champions, opinion leaders, and change agents
Capacity	Necessary skills to engage and act on the innovation.	3	Necessary skills

**Table 5:** Dissemination strategy constructs, suggested definition, frequency of construct across frameworks, and other names in the literature

Strategy construct	Suggested definition	Total frequency	Other names in the literature
Identify the quality gap	Synthesize and critically appraise the information	6	Identify, review, select knowledge
Assess dissemination determinants	Examine barriers and facilitators for the spread of information	3	Assess barriers to knowledge use
Assess determinants of innovation uptake	Examine what contextual conditions are necessary to achieve the outcomes from the innovation uptake	2	Assess barriers to innovation uptake
Adapt the information to the context	Connect the information and the medium used to share the information with existing priorities and responsibilities of stakeholders	5	Adapt information to the context
Funding	Changes in the financial structure	5	Funding
Policy change	Changes in policy	5	Policy change
Monitoring and evaluation	Monitoring and evaluation of dissemination milestones and goals	10	Monitoring and evaluation
Sustain knowledge use	Examine determinants for sustained use of knowledge	2	Sustain knowledge use
Increase audience’s skills	Increase audience’s skills to uptake the innovation	6	Increase skills of end-users, coaching, academic detailing, group discussion, facilitation

**Table 6:** Dissemination outcome constructs, suggested definition, frequency of construct across frameworks, and other names in the literature

Outcome construct	Suggested definition	Total frequency	Other names in the literature
Awareness	The user/audience being cognizant of the information or communication	8	Awareness
Reception	The audience must give attention to reading the incoming message	2	Reception
Persuasion	When an individual forms a favorable or unfavorable attitude towards the innovation	3	Persuasion
Emotion reactions	Emotional state at the time of the message encounter and by feelings induced by the message	3	Affective reactions
Decision	Choices to accept or reject an innovation that are made by an individual independent of the decisions of the other members of the system	5	Decision, rationale
Knowledge gained	Knowledge gain when an individual or group of people learn about the innovation	3	Knowledge gained
Knowledge utilization	Knowledge and skills to engage with the innovation	11	Knowledge utilization
Changes in policy	Structural changes to facilitate the uptake of the innovation	8	Changes in policy, economics
Adoption	The individual or organization engages in a number of activities that will lead to the research evidence being integrated into clinical practice and/or policy decisions	4	Adoption
Fidelity	To what extent were the various intervention components delivered as intended (in the protocol)	1	Fidelity, adherence
Confirmation	When an individual or an organization seeks reinforcement of an innovation decision that has already been made	2	Confirmation
Accountability	Establishing clear responsibilities and expectations for stakeholders	3	Accountability
Impact	When the uptake of the innovation has tangible benefits	5	Impact
Maintenance, long-term outcome	The extent to which a program or policy becomes institutionalized or part of the routine organizational practices and policies	3	Maintenance
Cost	Cost of the dissemination process	5	Cost

Table 3 shows the constructs that relate to the *dissemination processes*, i.e., the steps or processes through which dissemination happens. Seven constructs were categorized as processes: knowledge inquiry, knowledge synthesis, communication, interaction, persuasion, activation, and research transfer. That is, six frameworks suggest that the dissemination process starts with an inquiry of what type of information is needed to close the knowledge gap. Next, there is a process of gathering and synthesizing the information, including examining the context in which the information will be shared. After the information is identified and gathered, there is a process of communication, interaction with the information, and persuasion where the information is shared with the target users, where the users then engage with the information and activate towards action based on the information received. Finally, there is a process of research transfer, where the information sharing “becomes essentially independent of explicit intentional change activity.” [33]

Table 4 shows the 17 constructs categorized as *dissemination determinants*, which are constructs that reflect aspects that may facilitate or hinder the dissemination process. Determinants identified included content of the information, context, interpersonal networks, source of knowledge and audience, the medium of dissemination, opinion leaders, compatibility of the information with the setting, type of information, and capacity of the audience to adopt the innovation. Communication, the salience of communication, and users’ perceived attitudes towards the information were the most frequent constructs (*n* = 14 each), followed by context (*n* = 13), interpersonal networks (*n* = 12), sources of knowledge, and audience (*n* = 10 each).

Table 5 shows the nine constructs related to *dissemination strategies*, which are constructs that describe the approaches or actions to promote or support dissemination. Leeman and colleagues [34] conceptualize dissemination strategies as strategies that provide synthesis, translation, and support of information. The authors refer to dissemination as two broad strategies: developing materials and distributing materials. We identified several strategies related to the synthesis of information (e.g., identify the knowledge), translation of information (e.g., adapt information to context), and other constructs. Monitoring and evaluation were the most frequent constructs (*n* = 10), with identify the quality gap and increase audience’s skills next (*n* = 6).

Finally, Table 6 shows the *dissemination outcomes*, which are constructs related to the effects of the dissemination process. Fifteen constructs were categorized here, including awareness and changes in policy, decision and impact, adoption and cost, emotion reactions, knowledge gained, accountability, maintenance, persuasion, reception, confirmation, and fidelity. Knowledge utilization was the most cited construct across frameworks (*n* = 11), followed by awareness and change in policy (*n* = 8 each).

## Discussion

The goals of this study were threefold. First, we conducted a scoping review of the empirical literature to catalog the dissemination frameworks informing dissemination studies. Second, we compiled the definition of dissemination, and third, we cataloged and defined the constructs from the dissemination frameworks. During our review process, we found that clearly identifying dissemination studies was more complicated than anticipated. Defining the sample of articles to code for this study was a challenge because of the large variability of studies that use the word “dissemination” in the titles but that are actually scale-up or health communication studies.

The high variability in the definition of dissemination poses a challenge for the field because if we do not clearly define what we are doing, we are unable to set boundaries to distinguish dissemination research from other fields. Among the identified frameworks that defined dissemination, the definitions highlighted that dissemination involved the spread of something, whether knowledge, an innovation, or a program. Distinct from diffusion, several definitions described dissemination as an active process, using intentional strategies. Few definitions described the role of determinants, whether dissemination is a process or a discrete event, and what strategies and outcomes may be pertinent. Future work is needed to unify these distinct conceptualizations into a comprehensive definition that dissemination researchers can use.

While it is clear that dissemination differs from diffusion, as the latter has been considered the passive and “haphazard” spread of information [35], the distinction between dissemination and scale-up—as shown in the definitions identified in this study—is less clear. Some articles from our search not included in the review conceptualized dissemination as similar to scale-up. To clarify the distinction between dissemination and scale-up in our review, we used the WHO’s definition of scale-up [36] as “deliberate efforts to increase the impact of successfully tested health innovations to benefit more people and to foster policy and program development on a lasting basis.” In other words, based on these definitions, our team considered scale-up as referencing *active* efforts to spread *evidence-based interventions*, whereas diffusion is the *passive* spread of *information.* Dissemination, therefore, can be conceptualized as the *active* and *planned* spread of *information.*

**Fig. 2:**
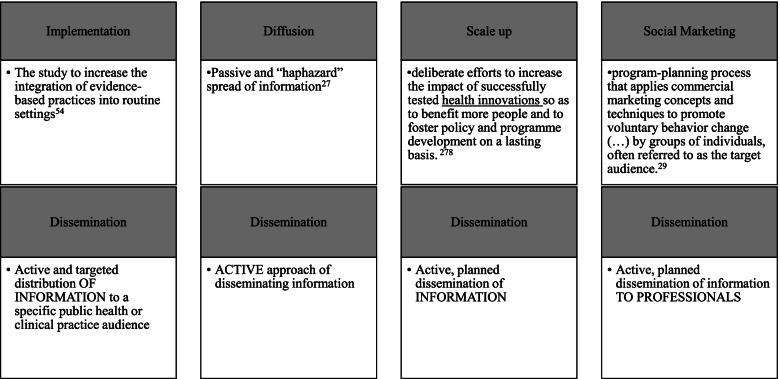
Proposed distinction of definitions between diffusion, scale-up, and dissemination

Another helpful component in distinguishing dissemination science from other sciences is related to the target audience. Brownson et al. [1] define dissemination as an “active approach of spreading evidence-based information to the target audience via determined channels using planned strategies” (p. 9). Defining the target audience in the context of dissemination is important because it may help distinguish the field from social marketing. Indeed, several studies we excluded involved sharing information with the public (e.g., increasing the awareness of the importance of sunscreen in public swimming pools). Grier and Bryant define social marketing as a “program-planning process that applies commercial marketing concepts and techniques to promote voluntary behavior change ( … ) by groups of individuals, often referred to as the target audience.” [37] The target audience in the context of social marketing, the authors explain, is usually considered consumers but can also be policymakers [37]. To attempt to delineate a distinction between these two fields, dissemination work has traditionally identified professionals (e.g., clinicians, public health practitioners, policymakers) as the target audience of dissemination efforts, whereas the target audience in social marketing is conceptualized as a broader audience. Figure 2 shows how we conceptualize the distinct components of dissemination research from other fields. Based on these distinctions, we propose the following coalesced definition for dissemination research to guide this review: *the scientific study of the targeted distribution of information to a specific professional person or group of professionals.* Clearly distinguishing dissemination from scale-up as well as health communication will help further advance the dissemination research field.

Our results show that of the empirical papers identified in this review, 51% used a framework to guide their study. This finding mirrors the suboptimal use of frameworks in the field of implementation research [38, 39], with scholars recently putting forth guidance on how to select and use frameworks to enhance their use in implementation research studies [6]. Similarly, we provide a catalog of dissemination frameworks and their constructs identified in dissemination studies. It is necessary to move the dissemination research field forward by embedding frameworks in dissemination-focused studies.

Some empirical papers included in our review used frameworks based on the knowledge translation literature. Knowledge translation, a field most prominent outside the USA, has been defined as “a dynamic and iterative process that includes the synthesis, dissemination, exchange and ethically sound application of knowledge to improve health, provide more effective health services and products, and strengthen the health care system” [40]. As such, it conceptualizes an interactive relationship between the creation and the application of knowledge. In the USA, however, researchers tend to conceptualize dissemination as a concept discrete from implementation and use the acronym “D&I” to identify these two fields.

While one could state that there is a distinct set of outcomes, methods, and frameworks between dissemination and implementation fields, previous scholars have cataloged [4] a continuum, from dissemination only” to “implementation only” frameworks. Consistent with this, our findings show that scholars have adapted implementation frameworks to fit dissemination outcomes (e.g., Klesges’ adaptation of RE-AIM [41]), while other frameworks have both dissemination and implementation components (e.g., integrated Promoting Action on Research Implementation in Health Services [i-PARIHS] [42]). Additionally, behavioral change frameworks (e.g., theory of planned behavior) were cataloged in our study as they were used in included articles. The use of implementation frameworks in studies identified here as dissemination studies highlights at least three potential hypotheses. One possibility is the use of implementation frameworks in dissemination studies is due to the underdevelopment of the field of dissemination, as shown in the challenges that we found in the conceptual definition of dissemination. We hope that, by clearly outlining a definition of dissemination, scholars can start to empirically examine whether there are distinct components between implementation and dissemination outcomes and processes.

The second hypothesis is that we still do not have enough evidence in the dissemination or implementation fields to be dogmatic about the categorization of frameworks as either “dissemination” or “implementation.” Until we have more robust evidence about what is and what is not dissemination (or other continua along which frameworks may be categorized), we caution against holding too firm to characterizations of frameworks [38, 43–45] Frameworks evolve as more empirical evidence is gathered [43, 45–49], and they are applied in different settings and contexts. We could hypothesize that it is less important, as of now, to categorize a framework as an implementation or dissemination framework and instead clearly explain why a specific framework was selected and how it is applied in the study.

Selection and application of frameworks in dissemination and implementation research is still a challenge, especially considering scholars may often select frameworks in a haphazard way [6, 50, 51]. While scholars have put forward some guidance to select implementation frameworks [6, 52], the challenge in the dissemination and implementation research fields is likely not only in the selection of the frameworks but perhaps more so in the misuse or misapplication of frameworks, theories, or models. A survey indicated that there is little consensus on the process that scholars use to select frameworks and that scholars select frameworks based on several criteria, including familiarity with the framework [50]. As such, Birken et al. [52] offer other criteria for the selection of frameworks, such as (a) usability (i.e., whether the framework includes relevant constructs and whether the framework provides an explanation of how constructs influence each other), (b) applicability (i.e., how a method, such as an interview, can be used with the framework; whether the framework is generalizable to different contexts), and (c) testability (i.e., whether the framework proposed a testable hypothesis and whether it contributes to an evidence-based or theory development). Moullin et al. [6] suggest that implementation frameworks should be selected based on their (a) purpose, (b), levels of analysis (e.g., provider, organizational, system), (c) degree of inclusion and depth of analysis or operationalization of implementation concepts, and (d) the framework’s orientation (e.g., setting and type of intervention).

More than one framework can be selected in one study, depending on the research question(s). The application of a framework can support a project in the planning stages (e.g., examining the determinants of a context, engaging with stakeholders), during the project (e.g., making explicit the mechanisms of action, tracking and exploring the process of change), and after the project is completed (e.g., use of the framework to report outcomes, to understand what happened and why) [6, 51, 53]. We believe that similar guidance can and should be applied to dissemination frameworks; further empirical work may be needed to help identify how to select and apply dissemination and/or implementation frameworks in dissemination research. The goal of this review is to support the advancement of the dissemination and implementation sciences by identifying constructs and frameworks that scholars can apply in their dissemination studies. Additional file 1: Tables S6-S9 show the frequency of constructs per framework, and readers can see the variability in the frequency of constructs per framework to help in their selection of frameworks.

A third hypothesis is that the processes of dissemination and implementation are interrelated, may occur simultaneously, and perhaps support each other in the uptake of evidence-based interventions. For example, Leppin et al. [54] use the definition of implementation based on the National Institutes of Health: “the adoption and integration of evidence-based health interventions into clinical and community settings for the purposes of improving care delivery and efficiency, patient outcomes, and individual and population health” [55], and implementation research as the study of this process to develop a knowledge base about how interventions can be embedded in practices. In this sense, implementation aims to examine the “how” to normalize interventions in practices, to enhance uptake of these interventions, guidelines, or policies, whereas dissemination examines how to spread the *information* about these interventions, policies, and practices, intending to support their adoption (see Fig. 1). In other words, using Curran’s [56] simple terms, implementation is about adopting and maintaining “the thing” whereas dissemination is about intentionally spreading information to enable learning about “the thing.” As Leppin et al. argue, these two sciences [54, 57, 58], while separate, could co-occur in the process of supporting the uptake of evidence-based interventions. Future work may entail empirically understanding the role of these frameworks in dissemination research.

This review aimed to advance a critical step in the dissemination literature by defining and categorizing dissemination constructs. Constructs are subjective, socially constructed concepts [59], and therefore their definitions may be bounded by factors including, but not limited to, the researchers’ discipline and background, the research context, and time [60]. This is evident in the constructs’ lack of consistent, clear definitions (see Additional file 1). The inconsistency in the definitions of the constructs is problematic because it impairs measurement development and consequently validity and comparability across studies. The lack of clear definitions of the dissemination constructs may be due to the multidisciplinary nature of the D&I research field in general [61, 62], which is a value of the field. However, not having consistency in terms and definitions makes it difficult to develop generalizable conclusions and synthesize scientific findings regarding dissemination research.

We identified a total of 48 constructs, which we separated into four categories: dissemination processes, determinants, strategies, and outcomes. By providing these categories, we can hope to help advance the field of dissemination research to ensure rigor and consistency. Process constructs are important to guide the critical steps and structure that scholars may need to take when doing dissemination research. Of note is that the processes identified in this study may not be unique to dissemination research but rather to the research process in general. As the field of dissemination research advances, it will be interesting to examine whether there are unique components in these process stages that are unique to the dissemination field. In addition to the process, an examination of dissemination determinants (i.e., barriers and facilitators) is essential in understanding how contextual factors occurring at different levels (e.g., information recipient, organizational setting, policy environment) influence dissemination efforts and impede or improve dissemination success [7]. Understanding the essential determinants will help to guide the selection and design of strategies that can support dissemination efforts. Finally, the constructs in the dissemination outcomes will help examine levels and processes to assess.

The categorization of the constructs was not without challenges. For example, persuasion was coded as a strategy (persuading) and as an outcome (persuasion). Likewise, the construct *confirmation* could be conceptualized as a stage [16] or as an outcome [19]. The constructs identified in this review provide an initial taxonomy for understanding and assessing dissemination outcomes, but more research and conceptualization are needed to fully describe dissemination processes, determinants, strategies, and outcomes. Given the recent interest in the dissemination literature [22, 63], a future step for the field is examining the precise and coherent definition and operationalization of dissemination constructs, along with the identification or development of measures to assess them.

### Limitations

A few limitations to this study should be noted. First, the search was limited to one bibliometric database and from journals publishing D&I in health studies. We limited our search to one database because we aimed to capture articles from Norton et al. [11], and therefore, our search methodology was focused on journals instead of on databases. Future work learning from other fields, and doing a broader search on other databases could provide different perspectives. Second, we did not include terms such as research utilization, research translation, knowledge exchange, knowledge mobilization, or translation science in our search, limiting the scope and potential generalizability of our search. Translation science has been defined as being a different science than dissemination, however. Leppin et al. [54], for example, offer the definition of translation science as the science that aims to identify and advance generalizable principles to expedite research translation, or the “process of turning observations into interventions that improve health” (see Fig. 1). Translation research, therefore, focuses on the determinants to achieve this end. Accordingly, Wilson et al. [7] used other terms in their search, including translation, diffusion of innovation, and knowledge mobilization and found different frameworks in their review. In their paper, Wilson and colleagues [7] provided a different analysis than ours in that they aimed to examine the theoretical underpinning of the frameworks identified by them. Our study is different from theirs in that we offer the definition of disseminating and a compilation of constructs and their definitions. A future study could combine the frameworks identified by our study with the ones identified by Wilson and colleagues and detail the theoretical origins of the frameworks, and the definitions of the constructs to support in the selection of frameworks for dissemination studies. Third, by being stringent in our inclusion criteria, we may have missed important work. Several articles were excluded from our scoping review because they were examining the spread of an evidence-based intervention (scale up) or of the spread of dissemination for the public (health communication). As noted above, however, clearly distinguishing dissemination from scale-up and from health communication will help further advance the dissemination research field and identify its mechanisms of action. Fourth, given the broad literatures in diffusion, dissemination, and social marketing, researchers may disagree with our definitions and how we conceptualized the constructs. Fifth, we did not code qualitative studies because we wanted to have boundaries in this study as it is a scoping study. Future studies could examine the application of frameworks in qualitative work. It is our hope that future research can build from this work to continue to define and test the dissemination constructs.

## Conclusions

Based on the review of frameworks and the empirical literature, we defined dissemination research and outlined key constructs in the categories of dissemination process, strategies, determinants, strategies, and outcomes. Our data indicate that the field of dissemination research could be advanced with a more explicit focus on methods and a common understanding of constructs. We hope that our review will help guide the field in providing a narrative taxonomy of dissemination constructs that promote clarity and advance the dissemination research field. We hope that future stages of the dissemination research field can examine specific measures and empirically test the mechanisms of action of the dissemination process.

## Supplementary Information

**Additional file 1: Table S1.** Definition of disseminations from frameworks. **Table S2.** Dissemination Process constructs, their definition, and frequency across frameworks. **Table S3.** Dissemination determinants constructs, their definition, and frequency across frameworks. **Table S4.** Dissemination strategy constructs, their definition, and frequency across frameworks. **Table S5.** Dissemination outcome constructs, their definitions, and frequency across frameworks. **Table S6.** Frequency of Process Constructs Across Frameworks. **Table S7.** Frequency of Determinant Constructs Across Frameworks. **Table S8.** Frequency of Strategy Constructs Across Frameworks. **Table S9.** Frequency of Determinant Constructs Across Frameworks. Preferred Reporting Items for Systematic reviews and Meta-Analyses extension for Scoping Reviews (PRISMA-ScR) Checklist [64–82].

## Data Availability

The datasets used and/or analyzed during the current study are available from the corresponding author on reasonable request.

## Abbreviations

D&I: Dissemination and implementation
i-PARIHS: Integrated Promoting Action on Research Implementation in Health Services
RE-AIM: Reach, Effectiveness, Adoption, Implementation, Maintenance
CFIR: Consolidated Framework for Implementation Research
