# Supplementary material for: A scoping review of frameworks in empirical studies and a review of dissemination frameworks
Source: Implement Sci. 2022 Aug 9;17:53. doi: 10.1186/s13012-022-01225-4 (PMC9361268; doi:10.1186/s13012-022-01225-4)
Supplement: Supplementary file 1 — Additional file 1: Table S1. Definition of disseminations from frameworks. Table S2. Dissemination Process constructs, their definition, and frequency across frameworks. Table S3. Dissemination determinants constructs, their definition, and frequency across frameworks. Table S4. Dissemination strategy constructs, their definition, and frequency across frameworks. Table S5. Dissemination outcome constructs, their definitions, and frequency across frameworks. Table S6. Frequency of Process Constructs Across Frameworks. Table S7. Frequency of Determinant Constructs Across Frameworks. Table S8. Frequency of Strategy Constructs Across Frameworks. Table S9. Frequency of Determinant Constructs Across Frameworks. Preferred Reporting Items for Systematic reviews and Meta-Analyses extension for Scoping Reviews (PRISMA-ScR) Checklist [64–82]. [file 13012_2022_1225_MOESM1_ESM.docx]

Table A1. Definition of disseminations from frameworks.

| **Frameworks** | **Definition of dissemination** |
| --- | --- |
| Framework 1 – Rogers’ Diffusion of Innovation^16^ | “Dissemination is the diffusion that is directed and managed Diffusion is the planned and spontaneous spread of new ideas.” (pg. 6) |
| Framework 2 - RAND model of persuasive communication and diffusion of medical innovation^63^ | “Dissemination and acceptance of medical technology assessments can be understood within the context of theories of diffusion of innovation and of persuasive communication.” (p.314) |
| Framework 3 - Effective dissemination strategies^23^ | “Dissemination is therefore seen as a process that aims to ensure that key messages are conveyed to specified groups via a wide range of methods such that it results in some reaction, some impact or implementation.” (p.70) |
| Framework 4 - Model for locally based research transfer development^22^ | “Transferring research to the users” (pg. 1008) |
| Framework 5 - Streams of policy process^21^ | Not explicitly defined |
| Framework 6 - Conceptual model of knowledge utilization^65^ | Not explicitly defined |
| Framework 7 - Conceptual framework for research knowledge transfer and utilization^66^ | Not explicitly defined |
| Framework 8 - Conceptualizing dissemination research and activity: Canadian heart health initiative^24^ | “Whereas some diffusion processes can be characterized  as passive or natural processes, others involve directed diffusion, or dissemination; that is, an active, deliberate, planned process to spread an innovation.” (pg. 271) |
| Framework 9 - Policy framework for increasing diffusion of evidence based physical activity interventions^25^ | “Dissemination is the set of planned, systematic efforts  designed to make a program or innovation more widely available; diffusion is the direct or indirect outcomes of  those efforts.” (pg. S35) |
| Framework 10 - Blueprint for dissemination^26^ | “The various factors that influence the spread of innovation are on a continuum between pure diffusion (in which spread occurs spontaneously through decen­tralized and informal efforts) and active dissemination (in which spread occurs purposefully through central­ized and formal efforts). This report focuses on active dissemination, that is, planned efforts to persuade targeted groups to adopt an innovation.” (pg. 2) |
| Framework 11 - Framework for knowledge translation^67^ | Not explicitly defined |
| Framework 12 – Knowledge to Action Framework^27^ | “The spreading of knowledge or research, such as is done in scientific journals and at scientific conferences.” |
| Framework 13 - RE-AIM^18,40^ | Not explicitly defined |
| Framework 14- Dobbins' Framework for the Dissemination and Utilization of Research for Health-Care Policy and Practice^19^ | “Dissemination research, defined as the study of the  processes and variables that determine and/or influence the  adoption of knowledge, interventions or practice by various  stakeholders…” |
| Framework 15 - Interactive Systems Framework^20^ | Not explicitly defined |
| Framework 16 - Network Theory^68^ | Not explicitly defined |
| Framework 17 - Affective Reactions Model^69^ | Not explicitly defined |
| Framework 18 - COM-B Model^70^ | Not explicitly defined |
| Framework 19 - Edquist's Model of Process and Product Innovation^71^ | SM 11-21: Not explicitly defined. |
| Framework 20 - Experimental Social Innovation and Dissemination ^28,32,72^ | *Fairweather, G.W., & Tornatzky, L. G. (1977). Experimental methods for social policy research. New York: Pergamon*  *Fairweather, G.W. (1967). Methods for experimental social innovation. New York: Wiley*  *Fairweather, G. W., & Davidson, W. S. (1986). An introduction to community experimentation: Theory, methods and practice. New York: McGraw-Hill.*  the social processes by which an innovation is promoted so that it can be spread through a society (pg 209).  Not explicitly defined |
| Framework 21 - Information Processing Model^73^ | Not explicitly defined |
| Framework 22 - Interaction Model of Research Use^30,31^ | 22a: “…Dissemination is deemed to occur when a potential user becomes aware of the research results. This model explains knowledge utilization with the recourse to two determinants: the types of research results and the dissemination effort.” ^48^  22b: Not explicitly defined |
| Framework 23 - Kumagai's Conceptual Framework for the Use of Illness Narratives in Medical Education^74^ | Not explicitly defined |
| Framework 24 - Medical Research Councils’ Theory of Change^75^ | Not explicitly defined |
| Framework 25 - Miller’s Framework for Clinical Assessment^76^ | Not explicitly defined |
| Framework 26 - Physical Activity Policy Research Framework^77^ | Not explicitly defined |
| Framework 27 - Promoting Action on Research Implementation in Health Services (PARIHS)^41^ | Not explicitly defined |
| Framework 28 - SPIRIT Action Framework^78^ | Not explicitly defined |
| Framework 29 - Systematic Review of Dissemination Planning Frameworks and Strategies^7^ | “We define dissemination as a planned process that involves consideration of target audiences and the settings in which research findings are to be received and, where appropriate, communicating and interacting with wider policy and health service audiences in ways that will facilitate research uptake in decision-making processes and practice.” (pg. 2) |
| Framework 30 - Technology Acceptance Model^79^ | Not explicitly defined |
| Framework 31 - Thacker's Framework for Environmental Health Surveillance^80^ | Not explicitly defined |
| Framework 32 - Theory of Middle Managers’ Roles in Healthcare EBP Implementation^29^ | “Diffusing information: Middle managers disseminate facts, giving employees necessary information about innovation implementation.” (pg. 5) |
| Framework 33 - Theory of Planned Behavior^81^ | Not explicitly defined |
| Framework 34 - Weingarden's Stages of Implementation Model^82^ | Not explicitly defined |

Table A2. Dissemination Process constructs, their definition, and frequency across frameworks

| **Dissemination Process** | **Suggested definition** | **Definitions from frameworks** | **Frameworks that used the construct** | **Total frequency** | **Other names in the literature** |
| --- | --- | --- | --- | --- | --- |
| Knowledge Inquiry | Inquiry about the knowledge gap: examine what is known, who to approach, how to approach, why to approach stakeholders to achieve the change in the context | Represents the unmanageable multitude of primary studies or information of variable quality. This can be thought of as first-generation knowledge that is in its natural state and largely unrefined, like diamonds in the rough that is out there and that may or may not be easily accessed. ^47^  “Much dissemination activity stops before it even begins. One glaring deficit in the dissemination literature is answers to the basic question of how to make initial contacts with target organizations, people, or units. Thus, such basic questions as modality of approach, who to approach, intensity or approach, and their interrelations all need to be systematically explored through experiments” ^25,57,58^  to examine when and how decision makers use information, we need to understand how environment affects decision makers' processing of information ^48^  “At the start of the intervention development phase, ToC uses a participatory approach by bringing together a range of stakeholders (for example health service planners, healthcare workers and service users) to develop a ToC map and to encourage stakeholder buy-in to the project” (…) “stakeholders first agree on the real world impact they want to achieve. They then identify the causal pathways through which this change can be achieved in that context using the available resources“ ^62^  “The first priority is to develop better tools to assess the effects of policies and to guide policy development, and prioritize policy choices such as health impact assessment, cost effectiveness studies, and establishing policy surveillance systems and methods for rating their effectiveness. The second priority is to determine the effects of policies relating [the topic]” ^64^  Demonstrate a gap between what you and your colleagues think you are doing and what you are actually doing in clinical area that matters to your patients ^69^ | 12, 21, 24a, 26, 28, 36 | 6 | Knowledge Inquiry |
| Knowledge Synthesis | Synthezing the information to help make sense of the relevant knowledge. | “The process by which participants create and share information with one another in order to reach a mutual understanding” ^16 (p.18)^  Knowledge synthesis, or second-generation knowledge, represents the aggregation of existing knowledge. The process involves the application of explicit and reproducible methods to the identification, appraisal, and synthesis of studies or information relevant to specific questions. It is done to make sense of all the relevant knowledge. This knowledge often takes the form of systematic reviews, including meta-analysis and meta-synthesis. ^47^  The process of compiling and summarizing information about innovations is synthesis, and it is accomplished through a variety of methods: evidence synthesis, systematic review, integrative review, meta-analysis, review of literature, and state of the science review ^20^  A student, a resident, a physician *knows* what is required in order to carry out those professional functions effectively. There are many who appear to believe that this knowledge base is all that needs to be measured... They must develop, among other things, the skill of acquiring information from a variety of human and laboratory sources, to analyze and interpret these data, and finally to translate such findings into a rational diagnostic or management plan. It is this quality of being functionally adequate, or of having sufficient knowledge, judgement, skill, or strength for a particular duty that Webster defines as *competence*. ^63^  Middle managers integrate and interpret facts, making general information about innovation implementation relevant to unique organizations and employees. ^49^ | 1, 12, 15, 27, 34 | 5 | Knowledge Synthesis |
| Communication | The process of creating and sharing information with others. To distinguish communication from interaction, we conceptualize communication as a one way communication from researchers to the audience. | “The process by which participants create and share information with one another in order to reach a mutual understanding” ^16(p.18)^  In this stage, knowledge producers better discern what type of dissemination object is most relevant to users. ^44^  Show them that the guideline has closed the gap and made things better | 1, 8, 31 | 3 | Communication |
| Interaction | The process where there is an interaction and exchange of information between researchers and the audience. | Stage of the research transfer process where integration of the needs, knowledge, and skills of actors happens with the goal of mutual benefit; shared information can be debated and activities negotiated.^43^  A process through which the user/audience frames of reference (i.e., how they see the world), actions or impact are altered. ^50^  Should the audience come to the researcher or should the researcher come to the audience? To what extent, and in what ways, should the researcher continue to be available to the user after the project? ^52^  This is known as research utilization stage and is characterized by the individual or organization engaging in activities to transfer the research evidence into health-care policy and clinical practice. ^27^  the knowledge utilization depends on various disorderly interactions occurring between researchers and users rather than on linear sequences beginning with the needs of the researchers or the needs of the users. (…).. Researchers engage resources into dissemination efforts when they hold meetings to discuss the subject and scope of their projects with users, to discuss results ^48^  interaction between researchers and decision makers ^60^  Interaction, collaboration and communication with researchers through events, projects, networks, committees, etc ^65^ | 4, 6, 11, 14, 24a, 24b, 30 | 7 | Interaction |
| Persuading | The process of proactively communicating the dissemination object, including adding components such as quality gap and value added to the dissemination object. | Stage of the research transfer process where there are mechanisms in place for actors (e.g., researchers and community-based agencies) to more proactively communicate about their research needs and share knowledge and skills. ^43^  (..) before the target unit can make a verbal decision to adopt an innovation, there must occur a process of cajolery, “unfreezing, coercion, accompanied by reinforcement” ^25 (p. 78)^ | 4, 21 | 2 | Persuading |
| Activation | When the audience starts to act based on the dissemination object received. | Once persuaded, the user must proceed to action. ^25^  Argumentation is the primary way members of the policy community (inc. academics, researchers) prioritize policy proposals. ^21^ | 5, 21 | 2 | Activation |
| Research transfer | When the dissemination object received becomes independent of the agent and is transferred to the audience. | The innovation-decision process is “the process through which an individual (or other decision-making unit) passes from first knowledge of an innovation, to the formation of an attitude toward the innovation, to a decision to adopt or reject, to implement and use of the new idea, and to confirmation of this decision” ^16^  A process where there is interface between decision-makers and their research needs and the interest and expertise of researchers. Consists of three continuous, sequential phases--awareness, communication, and interaction—that correspond to increasing level of dissemination effectiveness. ^43, 27^  During the comprehension stage, the receiver interprets the literal content, draws connection between the message and previous knowledge, and attaches meaning to the message ^54^  “At some point during the change process further adoption of an innovation becomes essentially independent of explicit intentional change agent activity” ^25(p.79)^ | 1, 4, 14, 17, 21 | 5 | Research transfer, the innovation-decision process, Diffusion |

Table A3. Dissemination determinants constructs, their definition, and frequency across frameworks

| **Determinant Constructs** | **Suggested Definition** | **Definitions from frameworks** | **Frameworks** | **Total frequency** | **Other names in the literature** |
| --- | --- | --- | --- | --- | --- |
| Source of knowledge | The individual or unit that delivers the dissemination object. | The individual or other unit of adoption that has the knowledge, or has experience using the innovation ^16^  Originator of the message ^41^ or knowledge ^51^ distinction made between sources of information (affects awareness) and influence/persuasion (affects decision to use) ^41^.  Professional sources (literature and colleagues) play a predominant role in a people’s decisions to act. Highly respected individuals seem to legitimate the translation of knowledge into practice. ^41^  The importance of the characteristic of the source (e.g., professional affiliation) will depend on the topic and target audience ^42^  Decision-making interest, decision-makers’ participation ^43^  Credible source ^51^, ^45^  The producer who transfers the dissemination product to users. ^44^  Describe the participation rate and characteristics of those delivering the intervention. State the population of intervention agents that one would see eventually implementing the program and how the study interventionists compare to those who will eventually deliver the intervention. ^33^  Establish that the guideline comes from a reputable source ^40^ | 1, 2, 3, 4, 7, 8, 9, 13b, 14, 31 | 10 | Type of source, Originator of the message or knowledge, Decision makers, Intervention agents, Interventionists |
| Medium of communication | The means (form) by which the dissemination object is shared. | The means by which messages get from one individual to another ^16,20^  Medium of presentation or means of communication through which information is provided ^41^.  The medium needs to be consistent with what is known about the target audience (e.g., books, email discussion lists, etc). It may be important to use different mediums ^42^  Knowledge broker^51^  [Not explicitly defined, implied.] Means by which the dissemination object is transferred to the user; transfer strategy. ^44^  What is the mode or interaction: written or oral, formal or informal? What is the groups’ preference regarding length? ^52^  number of innovation characteristics are known to be associated with the diffusion of innovations ^27^  “One of the ways in which meaning is learned is through stories. In fact, in the context of human history and culture, stories arguably represent the most effective vehicle that human beings use to communicate the meaning of an existentially important experience to one another” ^61^  Middle managers disseminate facts, giving employees necessary information about innovation implementation. ^49^ | 1, 2, 3, 7, 8, 11, 15, 25, 34 | 9 | Type of communication channel, Medium of communication, Knowledge broker |
| Content of communication | The content of the message sharing the dissemination object. | “An innovation is an idea, practice or object that is perceived as new by an individual or other unit of adoption” ^46(p12)^  The content of the recommendations being disseminated ^21, 22 23,43,46,50,52^  Policy “proposals that fail to meet these criteria—technical feasibility, value acceptability within the policy community, tolerable cost, anticipated public acquiescence, and a reasonable chance for receptivity among elected decision-makers—are not likely to be considered as serious, viable proposals” ^21(p131)^  Some define it as research results, whose characteristics can include study size, methodological adequacy, and clear decision of objectives ^50^.  Concepts; skills or capacity; tools, innovative practices, programs, or interventions that are either new or perceived as such by potential users ^44^  Rigorous evidence, compelling idea ^51^ Is unambiguous? Is the research consistent and what is the quality of the research? Is the research fragmented or broad and synthetic in focus? Is it action-oriented? ^52^  Report the extent to which different components of the intervention are delivered (by different intervention agents) as intended in the protocol. ^33^  “Policy provides an organizing structure and guidance for collective and individual behavior. It may be defined as legislative or regulatory action taken by federal, state, city, or local governments, government agencies, or nongovernmental organizations such as schools or corporations. Policy includes formal and informal rules and design standards that may be explicit or implicit. (..)Policy may be conceptualized at three levels reflecting social and political commitment: (1) Formal written codes, regulations, or decisions; ^43^ Written standards that guide choices; ^23^ Unwritten social norms that influence behavior” (p. S22) ^64^  “successful implementation can be explained by a function of the relationship between three elements: evidence, context, and facilitation. (…)he evidence is scientifically robust and matches professional consensus and patient needs (“high” evidence)”^34^  As a property of the framework: “Have a clearly articulated purpose and identify the foci for change in the individual, the organisation and more widely” ^65(p148)^  The guideline should contain evidence grading and evidence tables; key recommendations should be clearly based on evidence ^40^ | 2, 3, 4, 5, 6, 7, 8, 10, 11, 13b, 28, 29, 30, 31 | 14 | Type of message content, format, dissemination object, innovation |
| Audience | Person or group receiving the dissemination object. | Another individual or other unit that does not yet have knowledge of, or experience with the innovation ^16^  Those receiving the communication; can group using personal characteristics (e.g., age, education), typical speed of awareness or decision to use new ^43^  Users are not to be viewed as passive recipients but as people who are active in determining how the information will be used or ignored. May consider involvement of the end users as part of the process to accelerate process of dissemination. Other considerations include density of the target user network, hierarchical relationships ^23^.  The degree of the involvement of end users or advocacy may be an important factor. Need to think who benefits from the information and for what purpose. Engagement of the community in the research process is key ^22^  Decision-makers; can group based on decision-making style or participation. The user’s interest in the process, the decision-making orientation to the usefulness (or non-usefulness) of policy analysis and their participation (or lack thereof) in the development of the analysis. Importance of examining users characteristics (age, education, experience) and user predispositions (attitudes) towards policy analysis ^50^  Potential users of the dissemination object; can group based on needs, concerns, and priorities, which may determine what is produced (e.g., user demand-based adaptation of the dissemination object ^44^  Types can include public constituencies, advocates, regulators, and private sector audiences ^45^, providers (e.g., medical professionals) ^52^. Need to examine the size of the group and how centralized is the group ^52^  Reach refers to the percentage and risk characteristics of persons who receive or are affected by a policy or program. Reach (as well as adoption) also concerns the characteristics of participants. ^18^ Reach (individual level) – what percentage of potentially eligible participants were excluded, took part, and how representative they were? ^33^  Adoption (setting/agency level) - What percentage of settings and intervention agents within these settings (e.g., schools/educators, medical offices/physicians) (a) were excluded, (b) participated, and (c) how representative were they? ^33^  “Different sources of evidence will be valued in different ways by different groups of people” ^34^ | 1, 2, 3, 4, 6, 8, 11, 13a, 13b, 29 | 10 | Type of user/audience/recipient/decision maker |
| Type of innovation | The type or characteristics, and value added of the innovation that is being communicated | Can be distinguished between material goods (i.e., introduction of a new good) and process (i.e., introduction of a new method or procedure) ^56^  Not only have some types of information assumed more importance than others in potential users' perceptions and, subsequently, in problem solving (Schneider, 1986), but also past studies often refer to "use" as if there are no significant differences among the various types of information that might be used.^60^  “we identified three different strands of “evidence” that can be used in clinical decision making—research, clinical experience, and patient preferences. Located on a continuum of high to low, “high” research evidence was presented as systematic reviews and randomised controlled trials (RCTs) and “low” as anecdotal and descriptive information. Similarly, patient preferences were located on a high to low continuum where “high” was indicated by a partnership approach to decision making and “low” by a lack of involvement. It was suggested that, to maximise the uptake of evidence into practice, evidence on all three continua needs to be located towards “high” on these dimensions” ^34(p175)^ | 20, 24b, 29 | 3 | Type of innovation |
| Complexity of the innovation | The degree of complexity of an innovation being communicated. | “The degree to which an innovation is perceived as difficult to understand and use” ^16(p16)^  Complexity represents the degree to which an innovation is perceived as difficult to understand and use ^19^  We hypothesize that as the number of publications increases, researchers have, as byproducts, more research results available for use by practitioners and professionals ^48^  We hypothesize that as the number of publications increases, researchers, have, as byproducts, more research results available for use by practitioners and professionals ^65^  Perceived ease of use (EOU) refers to the degree to which the prospective user expects the target system to be free of effort. ^66^ | 1, 14, 24a, 32 | 4 | Complexity of the innovation |
| Timing of dissemination object | The speed and distance of the spread of the dissemination object. | “The time dimension is involved in diffusion in ^16^ the innovation-decision process by which an individual passes from first knowledge of an innovation through its adoption or rejection, ^43^ the innovativeness of an individual or other unit of adoption (that is, the relative earliness/lateness with which an innovation is adopted) compared with other members of a system, and ^23^ an innovation’s rate of adoption in a system, usually measured as the number of members of the system who adopt the innovation in a given time period” ^16(p20)^  “The traditional peer review research article may take considerable time to be published, yet the full benefits of research will only be realised if the results can be expeditiously transferred to those who need them” (Buxton and Hanney, 1996) ^43^  The density, quality and characteristic of the network of the end users can affect speed and distance of the information distribution ^23^.  Importance of the “policy windows, the opportunities for action on given initiatives” which “stay open for only short periods” (p. 166). Policy entrepreneurs must have their proposals ready and problem documented to take advantage of both predictable and unpredictable policy windows. ^23^  Report specific amounts of time, costs, or both, required to deliver the intervention. ^33^ | 1, 2, 3, 5, 13b | 5 | Timing of information spread |
| Urgency of the innovation | The urgency related to the innovation; how immediate is the need to disseminate the dissemination object about this innovation. | Concern that technology can become outdated very quickly, therefore there is a need to balance the perceived benefit of enhanced communication and information access with the expected continued pressures on service budgets ^43^  Immediate decision needed ^50^  Examine the pace of the work of the group receiving the information. Does the research suggest an immediate application? ^52^  insufficient time to implement new ideas, insufficient time to review literature, and a lack of administrative support for implementing decisions that are based on research evidence ^19^  the more time the receiver spends considering the message and its supporting rationale, the more likely the message is to be accepted and recalled later. (…) the more time the received spends in generating counterarguments, the more likely the respondent will remember rejecting the message ^54^  “The researchers who painstakingly developed the social innovation in the first instance are the only persons with sufficient knowledge from their own experiences to adequately describe and activate the new social model. Therefore, it is clearly incumbent upon the initial program development researchers to spend whatever time is required in either direct dissemination strategies or in the packaging of the innovation in such a manner that adequate dissemination can proceed” ^25(p73)^  as policy issues change rapidly, decision makers are more likely to use information. That is, when facing unfamiliar policy problems, decision makers need more information to reduce the unfamiliarity or uncertainty of the problems and are, thus, more likely to use information in making decisions ^60^ | 2, 6, 11, 14, 17, 21, 24b | 6 | Urgency of the innovation |
| Triability of an innovation | The degree to which an innovation can be implemented on a limited basis. | “The degree to which an innovation may be experimented with on a limited basis.” ^16( p.16)^  Characteristics of the innovation can include relative advantage, compatibility, simplicity, trialability, and observability ^46^  the extent to which the innovation can be implemented on a small scale to determine its advantages or disadvantages (…). Another aspect of trialability includes “bandwagon pressures.” The literature suggests that organizations imitate other organizations that are proximate, either geographically or in their communication networks, and will adopt innovations when other organizations have adopted innovation ^19^ | 1, 10, 14 | 3 | Trialability of the innovation |
| Observability of the results | The degree to which the uptake of the innovation yields observable results. | “The degree to which the results of an innovation are visible to others” ^16^  Characteristics of the innovation can include relative advantage, compatibility, simplicity, trialability, and observability ^46^ | 1, 10 | 2 | Observability of the innovation’s results |
| Salience of the innovation | The relevance of the innovation to the audience. | “The perceive newness of the idea for the individual determines his or her reaction to it.(…)”^16(p12)^The relative advantage of an innovation is “the degree to which an innovation is perceived as better than the idea it supersedes (p.15)  Issue salience ^22, 50^  Policy alternatives can be “elevated on the governmental agenda because they can be seen as solutions to a pressing problem” (p. 172) within the problem stream. ^23^  The relevance of research. The relative weight of the message can be based on a ‘truth test’ about the research quality and conformity to user expectations (e.g., compatibility with knowledge, experience and values), a ‘utility test’, related to the action orientation from the research and challenge to the status quo; and the overall relevance of the organization’s needs ^22^  Rigorous evidence ^51^. Types of evidence: evidence of need established a health behavior problem as a public health priority, and includes the size, severity and cost of the problem. Evidence of demand reference key constructs from marketing and diffusion of innovation ^45^  Characteristics of the innovation can include relative advantage, compatibility, simplicity, trialability, and observability ^46^  Is unambiguous? Is the research consistent and what is the quality of the research? Is the research fragmented or broad and synthetic in focus? Is it action-oriented? ^52^  For what purpose does the group use the information? How relevant is the research to the group? ^52^  Even if individuals are exposed to an innovation, this exposure will have little effect unless the innovation is perceived as relevant and consistent with the attitudes of the individual as well as his/her organization. ^19^  Relative advantage is defined as the degree to which an innovation is perceived as better than the idea it supersedes and can be measured in economic terms, social prestige, satisfaction, and savings in time and effort ^19^  When information about innovations is accessible, user friendly, and clearly demonstrates the utility of the innovations ^20^  The content of the message must be perceived as personally relevant if it is to be considered on its merits. If it is perceived as highly relevant, the receiver will concentrate on issues central to the argument ^54^  Examining decision makers' needs for information or perception of decision making is important because information is usually acquired or disseminated on the basis of some real or perceived need ^60^  We identified three different strands of “evidence” that can be used in clinical decision making—research, clinical experience, and patient preferences ^34^  Perceived usefulness (U) is defined as the prospective user's subjective probability that using a specific application system will increase his or her job performance within an organizational context. ^66^ | 1, 4, 5, 6, 7, 9, 10, 11, 14, 15, 17, 24b, 29, 32 | 14 | Salience, evidence of need and demand, relative advantage of the innovation |
| Users’ perceived attitude towards the innovation | A more general concept than the salience of innovation, related to the audience’s perception of the process of innovation development (research) and the receptivity of the innovation. | Perceived attributes of an innovation, such as its relative advantage and compatibility. The experience with one innovation can affect the individual’s perception of the next innovation 16  A key barrier in dissemination can be negative attitude towards research; dissemination will not succeed if there is no support of research ^23^.  May distinguish between “curiosity” about research and “need” for research. Need to break away from the protective mind of “we have always done it that way” ^22^  Two communities theory suggests that direct use of policy research by decision makers is not likely because of the competing worldviews and belief systems of researchers and policy makers. Importance of political perspectives and values (e.g., individualism) in the acceptance of the information ^50, 52^  Ongoing change, receptivity ^51^, attitudes toward the innovation, individual concerns and motivations, the need for new solutions ^44^  Audience perceptions of the information determine whether it will seen as feasible, acceptable and compatible with the context ^45,52^  Many health-care practitioners and decisionmakers perceive research findings as not relevant to their practice and/or decision needs ^19^  Attitudes change in response to new beliefs or through a shift in the importance attached to existing beliefs ^54^  When faced with problems that are familiar and unambiguous, decision makers will use little information. ^60^  We can hypothesize that the more decision makers have negative attitudes toward information, the less they use it. The negative attitude toward information also indirectly affects use of information in combination with need for information and interaction between researchers and decision makers. ^60^  Different sources of evidence will be valued in different ways by different groups of people—for example, research evidence can be counter to patient preferences... The framework therefore now acknowledges that different types of research evidence are needed to answer different clinical questions. What is critical to implementation is that well conceived, designed, and conducted research is drawn upon, whether quantitative or qualitative ^34^  Extent to which the individual believes: (a) that use of research in policy/program work is important and valuable, and (b) that research use is valued in their organization ^65^  The person's attitude toward using the system ^66^ | 1, 3, 4, 6, 11, 7, 8, 9, 14, 17, 24b, 29, 30, 32 | 14 | Users’ attitude towards research and the innovation |
| Compatibility of the innovation with the setting | The degree to which an innovation is consistent with the context. | Some innovations may be desirable for one adopter, but undesirable for another adopter whose situation differs. Compatibility is the “degree to which an innovation is perceived as being consistent with the existing values, past experiences, and needs of adopters” ^53(p15)^  Characteristics of the innovation can include relative advantage, compatibility, simplicity, trialability, and observability ^46^  Perceptions regarding the innovation are determined by complex interactions among characteristics of the innovation, organization, environment and individual. ^19^  Compatibility refers to the degree to which an innovation is perceived as being consistent with the existing values, needs and past experiences of potential adopters. (…) The diffusion research demonstrates that organizational context has a major influence on decisionmakers’ and practitioners’ innovation behavior ^19^  A message is more readily integrated if the content with previous beliefs or if it represents completely new knowledge ^54^ | 1, 10, 14, 17 | 4 | Compatibility of the innovation with the setting |
| Context | Settings in which communications are received and potential adoption occurs. | Settings in which communications are received and potential adoption occurs. ^43,44,51^  Organizational context: number of providers/colleagues, size of organization, cosmopolitan orientation of organization, amount of staff training, political feasibility, availability of resources for implementation, centralization and formalization of decision-making, structural characteristics, operating rules, norms, internal champions, organization climate, the types of clients served ^22,43,44,46^. Also, previous experience with similar innovation, and the existence of innovation department ^44^  Societal issues and priorities, manifested in public opinion and mass media ^44^  Importance of examining the formal and informal structures of the user group; the political climate surrounding the group ^52^  Research publications do not reach a wide audience within the practice field and typically do not address the priorities of practitioners, who must consider the fiscal and political context within their communities, along with potential program effectiveness. In addition, gathering and integrating information on innovations has its own challenges, particularly given the interdisciplinary nature of research on prevention ^20^  “(…) there is a strong concern for such issues as disparity in cultural background, cosmopoliteness, and disparities in norms and values between the change agent and the target of change” ^25(p74)^  Use of knowledge is increased when the researchers focus their projects on the needs of users instead of focusing them only on the advancement of scholarly knowledge.(…) To identify the contextual factors of the users that influence knowledge utilization is a paramount task ^48^  In relation to information processing in policymaking, some scholars indicate that a key variable determining how effectively decision makers process and utilize information is organizational norms or structures ^60^  “we broadly interpret “environment” to include the social, cultural, physical (built and natural) and communications environments.” (p. S23) ^64^  The term “context” is used to refer to the environment or setting in which people receive healthcare services or, in the context of getting research evidence into practice, “the environment or setting in which the proposed change is to be implemented.” In its most simplistic form, the term here means the physical environment in which practice takes place. Such an environment has boundaries and structures that together shape the environment for practice. ^34^  Systems to support research use such as systems for identifying, collating and disseminating relevant research ^65^  Middle managers’ commitment to healthcare innovation implementation contributes to an organization’s implementation climate–employees’ shared perceptions of the extent to which innovation implementation is rewarded, supported, and expected ^49^  A strong implementation climate promotes implementation effectiveness–consistent, high-quality innovation use ^49^ | 2, 7, 8, 10, 11, 15, 21, 24a, 24b, 28, 29, 30, 34 | 13 | Context |
| Interpersonal Networks | Large umbrella term that includes the relationship between the audience members, its structure and its quality. | Social prestige as a factor that affect the dissemination of an innovation. Homophily is the degree to which two or more individuals who interact are similar in certain attributes (e.g., beliefs, education), whereas heterophily is the degree to which the individuals who interact are different in certain attributes. ^16^  The structure of the networks matters in the diffusion of innovation. ^16^  Interpersonal networks as a key activator of adoption of the innovation. Interpersonal channels involve face-to-face exchange between two or more individuals. ^16^  Source is more influential than the message, importance of known and trusted sources. It is important to consider multi-disciplinary research teams. Ensuring multidisciplinary group in the team may benefit alliances and trustworthiness of the source. ^23^  Importance of the quality of the network of end users (research relationships), amount of conflict ^22^  Fragmentation in the policy community (e.g., tightly knit community versus having many diverse elements) corresponds to more fragmented policy, less coherence, and more instability. ^23^  Relationship between producers and users ^44^  Social support ^51^, need to examine the groups’ attitudes towards decision making, how much conflict surrounds the issue ^52^.  How much trust and rapport exist between the research team and the user group? How much would the user group interact from the beginning with the research and will that representation remain throughout the project? ^52^  Perceptions regarding the innovation are determined by complex interactions among characteristics of the innovation, organization, environment and individual. ^19^  Environmental factors associated with the diffusion of innovations include collaboration among community networks (network embeddedness) ^19^  Build awareness of the innovation through personal and professional relationships ^53^  Paying more attention to the linkage mechanisms could increase utilization of social science knowledge ^48^  “the development of physicians is shaped by a hidden curriculum, which, through role modeling, informal conversations, expectations, and social norms, exerts a powerful influence— both beneficial and pernicious— on the attitudes, values, and behavior of physicians-in-training” (p.655) ^61^  Relationships (both formal and informal) between the policy/program organisation and researchers or research institutions ^65^ | 1, 3, 4, 5, 7, 8, 11, 14, 16, 24a, 25, 30 | 12 | Influence, quality of relationships, interpersonal channels, trustworthiness, linkage mechanisms |
| Opinion Leaders and Change Agents | Opinion leadership is the degree to which an individual is able to influence other individual’s attitudes. Change agent is an individual who influences client’s innovation-decisions in a certain direction and speed | Opinion leadership is the degree to which an individual is able to influence other individual’s attitudes. Change agent is an individual who influences client’s innovation-decisions in a certain direction and speed. ^16^  Invisible support of leaders, champions ^44^  Individuals have the ability to shape their organizations and environments just as organizations and environments shape individual behavior ^19^  County staff (with formal titles of ‘‘agent,’’ ‘‘advisor,’’ or ‘‘educator’’) serve as the intermediaries between the state office and the community-based policy makers and service providers. (…) Garner the support of influential persons who are willing and able to advocate for the innovation ^53^  We can hypothesize that information sources (here internal sources) directly affect use of information. That is, the more information that comes from their own agencies, the more policymakers are likely to use it ^60^  Leaders who model and support research use in policy/program agenda setting, development, implementation or evaluation ^65^  Middle managers justify innovation implementation, encouraging employees to consistently and effectively use innovations. ^49^  A large staff is not necessary; a clinic or practice can probably carry out the adaptation on its existing computer system. A team leader (usually a physician) is appointed and given some training and the support of a facilitator. A team of 5 to 10 is good (…) The best teams gave vigorous transforming leadership, with “champions” and “cheerleaders”, including a content-expert champion of the initial guideline, a mentoring leader (“a champion who grew up” after having successfully launched several projects), and a “godfather” (a leader who providers organizational leverage and support) (p.553) ^40^ | 1, 8, 14, 16, 24b, 30, 34, 31 | 8 | Champions, Opinion Leaders and Change Agents |
| Capacity | Necessary skills to engage and act on the innovation. | “In addition to having readily available, user-friendly information on innovations, adoption and implementation of innovations requires the development and support for new skills for innovation use through specialized training, monitoring of fidelity/adherence, and coaching and/or supervision” ^20^  Capability is defined as the individual’s psychological and physical capacity to engage int he activity concerned. It includes having the necessary knowledge and skills ^55^  Knowledge and skills in the actions to engage with and apply research in policy/program agenda setting, development, implementation or evaluation ^65^ | 30, 15, 18 | 3 | Necessary skills |

Table A4. Dissemination strategy constructs, their definition, and frequency across frameworks

| **Strategy Construct** | **Suggested definition** | **Definitions from frameworks** | **Frameworks** | **Total frequency** | **Other names in the literature** |
| --- | --- | --- | --- | --- | --- |
| Identify the quality gap | Synthesize and critically appraise the dissemination object | Once the relevant research is identified, it is then critically appraised to determine its validity and usefulness for the problem at hand. ^47^  Translate the scientific research evidence into a small number of relevant and usable key messages, followed by efforts to change clinical practice or implement policies based on the research evidence ^19^  State staff collaborate with university professors to conduct, synthesize and translate research findings ^53^  “we aim for the creation of reflective discussion to explore the insights the students have gained from the stories of their volunteers. Mezirow(p11) defines reflective discourse as a “critical assessment of assumptions [that] leads towards a clearer understanding by tapping collective experience to arrive at a tentative best judgment.” (p.656) ^61^  As a proposition of the framework: “Be informed by existing understanding of what influences the use of research in health policy, including descriptive models and empirical findings (Eccles et al., 2005; Rycroft-Malone and Bucknall, 2010), drawing on the widest possible range of social science” (p. 148)  Searching for or otherwise identifying research to inform policy/programs; Commissioning, collaborating in or undertaking new research or new analyses to inform policy/programs ^65^  Evaluating the quality of research and the generalizability and reliability of research results, including the applicability of identified research to local policy/program needs. ^54,65^ | 12, 14, 16, 17, 25, 30 | 6 | Identify, Review, Select Knowledge |
| Assess Dissemination Determinants | Examine barriers and facilitators for the spread of dissemination object | Assess for potential barriers that may impede or limit uptake of the knowledge so that these barriers may be targeted and hopefully overcome or diminished by intervention strategies. The barriers assessment should also identify supports or facilitators that can be taken advantage of. ^47^  To examine when and how decision makers use information, we need to understand how environment affects decision makers' processing of information ^48^  As a proposition of the framework: “. Provide an organising structure to build knowledge (Eccles et al., 2005; Gregor, 2002; Rycroft-Malone and Bucknall, 2010). It will generate testable hypotheses about the drivers of research” ^65^ | 12, 24a, 30 | 3 | Assess Barriers to Knowledge Use |
| Assess determinants of innovation uptake | Examine what contextual conditions are necessary to achieve the outcomes from the innovation uptake | Not only do county staff have to balance the agendas of state and local constituencies, but concurrently, they must also address the priorities of and provide program support to a wide array of diverse volunteers. If volunteers choose not to work with county staff, the program delivery system can crumble. ^53^  “Determining what contextual conditions are necessary to achieve the outcomes, what resources are required to implement the interventions, and how the program gains the commitment of those resources are crucial outputs of the process” ^62^ | 16, 26 | 2 | Assess Barriers to Innovation Uptake |
| Adapt the disseminatin object to the context | Connect the dissemination object and the medium used to share the dissemination object with existing priorities and responsibilities of stakeholders | A potential goal of synthesis involves identifying key characteristics and core elements of programs, processes, principles, or policies. Key characteristics are defined as the crucial activities and delivery methods for conducting an intervention that maybe tailored to the unique needs and contexts of different agencies and at-risk populations. (…) Translation is the process of converting (translating)scientific knowledge into practitioner-friendly products tobe used for implementation. ^20^  Connect the innovation with existing priorities and responsibilities of stakeholders. ^53^  Efforts to make reports more readable and easier to understand, efforts to make conclusions and recommendations more specific, more operational, efforts to focus on variables amenable to interventions by users, efforts to make reports more appealing ^48^  “This opportunity to develop perspective taking is particularly important for those students who have not had any personal experience” ^61^  A first decision following the choice of topic is whether to adopt or adapt an existing guideline or develop one’s own ^40^ | 15, 16, 24a, 25, 31 | 5 | Adapt information to the context |
| Funding | Changes in the financial structure | Changes in the source and amount ^22^; incentives ^52^  Environmental factors related to the socioeconomic infrastructure of the community have been shown to impact on such organizational structures as administrative intensity, formalization and centralization of decision-making ^19^  State staff do not dictate specific programs or activities at the county level. Rather, they establish priorities and direction by sponsoring and supporting small grant programs, conferences and convenings, curriculum and program development, and demonstration projects on new initiatives ^53^  Resources to support research use such as access to electronic journals, reference management software, staff with specialist research use skills ^65^ | 4, 11, 14, 16, 30 | 5 | Funding |
| Policy change | Changes in policy | Mandate (expand or limit); restructure (merge or decentralize); new provincial government (changes in priorities) ^22^  Implementation – Policy needs to become practice, Policy results, not inputs, is the proper standard ^50^  Procedural changes ^51^  Dissemination activities or strategies that focus on decision-making processes in government, nongovernmental organizations, and industry. These can be categorized into information, education, government regulatory process, corporate, NGO processes, lawsuits, and legislative actions ^45^  regulations and legislation; urbanization; peer pressure; competition among institutions to attract specialized professionals; and acquisition of prestige ^19^  Organisational policies that support or require research use in policy/program agenda setting, development, implementation or evaluation ^65^ | 4, 6, 7, 9, 14, 30 | 5 | Policy change |
| Monitoring and Evaluation | Monitoring and evaluation of dissemination milestones and goals | Program evaluation ^22^  Information about the operation or cost of an existing program can bring forth new problems in the problem stream, creating a policy window. ^23^  Goal setting; what gets measured gets done ^51^  A latter stage dissemination activity or strategy that involves monitoring and evaluation of milestones and goals ^46^  Elements that can be monitored include adoption ^50^ use ^45^ incentives used ^45,52^ changes in environments ^45^ and impact ^50^  Once the relevant research is identified, it is then critically appraised to determine its validity and usefulness for the problem at hand. (…) evaluate whether application of the knowledge actually makes a difference in terms of such things as health, practitioner, and system outcomes. ^47^  County staff seek to maximize the utilization of the research system, often by conducting local needs assessments and communicating best practices ^53^  “only programs that are evaluated as beneficial are used in the dissemination process. (…) All parameters must be specified” ^25(p73)^  “develop better tools to assess the effects of policies and to guide policy development, and prioritize policy choices such as health impact assessment, cost effectiveness studies, and establishing policy surveillance systems and methods for rating their effectiveness” ^64^  As a proposition for the framework: “Be capable of guiding the development and testing of specific and targeted interventions, including the generation of program logic models and the identification of proximal and distal outcomes and associated measures (Eccles et al., 2005; Gregor, 2002). An action framework creates the rationale for selecting particular intervention points and strategies” (p.148) ^65^  We propose three types of surveillance for use in environmental public health: hazard surveillance, exposure surveillance, and outcome surveillance ^67^  “(..) it takes about the same amount of time [4-6 months] to put a good measurement program, and then a few more months after to standardize the measurement across all clinics, and then it takes longer to get reliable information” (p.554) ^40^ | 4, 5, 6, 7, 9, 10, 12, 16, 21, 28, 30, 33, 31 | 10 | Monitoring and Evaluation |
| Sustain Knowledge Use | Examine determinants for sustained use of knowledge | Assess barriers to knowledge sustainability, tailor interventions to these barriers, monitor ongoing knowledge use, and evaluate the impact of initial use and sustained use of the knowledge. The sustainability phase should set in motion a feedback loop that cycles through the action phases. ^47^  “we jointly developed language and rationale for community design (..)While this plan is officially “advisory” it has the indirect effect of policy—an element in the approval of regional and local development plans is their compatibility with the state long range plan” ^64^ | 1, 28 | 2 | Sustain Knowledge Use |
| Increase audience’s skills | Increase audience’s skills to uptake the innovation | academic detailing, audit and feedback, and the use of opinion leaders; personal, one-to-one contact with the intended audience. ^19^  The individuals, organizations, and communities that carry out prevention delivery activities have varying levels of existing capacity (defined here as including both ability and motivation) to implement prevention. ^20^  General capacity-building is intended to enhance theinfrastructure, skills, and motivation of an organization, butit does not focus on a specific innovation ^20^.  Coach stakeholders as they directly experience and observe ^53^  Provide access to research, program models, and best practices through training and consultation ^53^  “small-group interactions through the instructors’ posing of questions or introducing examples of clinical situations which involve ethical conflicts, ambiguities, or controversies in order to stimulate thoughtful discussion. In this setting, the instructors are trained to gently challenge the students’ preconceptions and biases regarding illness, disability, medical care, and doctoring in order to stimulate an honest exploration of their own, each other’s, and society’s assumptions, values, and beliefs” ^61^  Facilitators have a key role to play in helping individuals and teams to understand what they need to change and how they need to change it in order to apply evidence to practice. ^63^  Facilitation is “a technique by which one person makes things easier for others”. ^34^ | 14, 15, 16, 25, 27, 29 | 6 | Increase skills of end-users, coaching, academic detailing, group discussion, facilitation |

Table A5. Dissemination outcome constructs, their definitions, and frequency across frameworks.

| **Outcome Construct** | **Suggested Definition** | **Definitions** | **Frameworks** | **Total frequency** | **Other names in the literature** |
| --- | --- | --- | --- | --- | --- |
| Awareness | The user/audience being cognizant of the information, dissemination object or communication | The user/audience being cognizant of the dissemination object or communication ^22,43,44,50,54^  The starting point of the process of research transfer ^22^ does not always result in next phases of research transfer (e.g., adoption) ^22,43^  Dissemination of research evidence occurs during the knowledge stage, when decisionmakers and practitioners become aware of new information (…) The extent to which an individual/organization becomes knowledgeable about the innovation ^19^  The mere reception of knowledge by the potential user does not imply its use ^48^  “A student, a resident, a physician knows what is required in order to carry out those professional functions effectively” (…) But as Alfred North Whitehead pointed out many years ago, there is nothing more useless than a merely well informed man. Tests of knowledge are surely important, but they are also incomplete tools in this appraisal if we really believe there is more to the practice of medicine than knowing” (p. 263) ^63^ | 2, 4, 6, 8, 14, 17, 24a, 27 | 8 | Awareness |
| Reception | The audience must give attention to the reading the incoming message | Utilization takes place when policymakers or advisers receive information (from Knott and Wildavsky, 1980, cited in 6)  “Once the communication is noticed, the message receiver must give attention to the reading the incoming message” ^54^ | 6, 17 | 2 | Reception |
| Persuasion | When an individual forms a favorable or unfavorable attitude towards the innovation | Persuasion takes place when an individual forms a favorable or unfavorable attitude towards the innovation ^16^  Could consist of distinct degrees, e.g., a policymaker reading, digesting, or understanding research studies. ^50^  Once an individual or organization becomes aware of an innovation, various factors (innovation, organization, environment and individual) contribute to the development of perceptions toward the innovation. This is referred to as the persuasion stage ^19^  Attitudes toward the innovation are formed and the individual/organization seeks to identify the consequences associated with adopting or not adopting the innovation ^19^ | 1, 6, 14 | 3 | Persuasion |
| Emotion reactions | Emotional state at the time of the message encounter and by feelings induced by the message | Information processing is affected by the individuals’ emotional state at the time of the message encounter and by feelings induced by the message. (…). The reaction to a persuasive message can be: pleasure, arousal and dominance ^54^  Motivation is defined as all those brain processes that energize and direct behaviour, not just goals and conscious decision-making. It includes habitual processes, emotional responding, as well as analytical decision-making ^55^  “The culture of medical training affects the meaning of medicine that individual physicians develop, and, in this environment, learning occurs on cognitive, affective, and experiential levels” (..) “very closely linked with their ability to foster perspective taking, narratives derive their power to communicate meaning in part through their ability to appeal to fundamental, universally held emotions—loss, anger, jealousy, joy, sadness, a sense of injustice, etc. This appeal gives hot cognitions their psychological and emotional force and may arouse a sense of urgency in the listener to address the causes of an individual’s suffering. In addition, when coupled with a moral dilemma or evidence of inequality, this type of hot cognition may inspire a commitment to understand and address causes of social injustice” (p. 655) ^61^ | 17, 18, 25 | 3 | Affective reactions |
| Decision | Choices to accept or reject an innovation that are made by an individual independent of the decisions of the other members of the system. | Decision occurs when an individual engages in activities that lead to a choice to adopt or reject the innovation ^16^  Optional innovation decisions are defined as choices to adopt or reject an innovation that are made by an individual independent of the decisions of the other members of the system. This means that the unit of analysis is at the level of the individual decision-maker. Collective innovation decisions, which are organizational choices to adopt or reject an innovation made by consensus among the members of a system, suggest that the unit of analysis is the organization. Finally, authority innovation decisions that are made by a relatively small number of individuals who possess power, status or technical expertise in a system suggest that the unit of analysis can be both the individual and the organization. ^19^  Acceptance is the initial step in incorporating information from the message into the receivers’ existing knowledge base. Acceptance include agreement to all or part of the message, with adjustment of prior beliefs if necessary and storage the message in memory ^54^  “Key beliefs that underlie why one outcome is an outcome for the next, and why you must do certain activities to produce the desired outcome. Can be based on evidence or experience” ^62^  People form intentions to perform behaviors toward which they have positive affect ^66^ | 1, 14, 17, 26, 32 | 5 | Decision, rationale |
| Knowledge gained | Knowledge gaine when an individual or group of people learn about the innovation | “Knowledge is gained when an individual (or other decision-making unit) learns of the innovation’s existence and gains some understanding of how it functions” (p.20)  Cognition – the policymaker must read, digest, and understand the studies. ^50^  At this stage the receiver determines whether the message represents new knowledge and whether it is consistent or inconsistent with prior beliefs ^54^ | 1, 6, 17 | 3 | Knoweledge gained |
| Knowledge utilization | Knowledge and skills to engage with the innovation | “Newness of an innovation may be expressed in terms of knowledge, persuasion, or a decision to adopt” ^16(p12)^  Practice acceptance of a recommended change; decision to use. The distinction between awareness and decision to act is important regarding sharing information compared to persuading for action ^43^  An effective research transfer should facilitate adoption, improve services. Distinction between using versus doing research ^22^  Effort – to make difference, information mush influence the actions of policymakers, who should fight for the adoption of study’s recommendations. Adoption – whether the information influence policy outcomes (from Knott and Wildavsky, 1980, cited in 6).  The use of the dissemination object, can be categorized into conceptual use, making the effort to use, procedural use, structural use ^51^.  During the decision stage, individuals and organizations engage in evidence-based decision-making activities that result in a decision to either adopt or reject an innovation (research evidence). ^19^  “certain stories stimulate self-reflection on one’s own perspectives, values, and biases and are all the more powerful in doing so because of their link with fundamental processes in learning and moral development. Both Piaget and Kohlberg believe that progress from one developmental stage to another is initiated by a cognitive disequilibrium, which occurs when an individual encounters unfamiliar or new ideas, values, perspectives, or circumstances” (p.656) ^61^  “The type of learning that the use of narratives attempts to enhance is, thus, fundamentally different than that involved in the teaching of the biomedical sciences. It is transformative: it consists of a process that involves learning on cognitive, affective, and experiential levels and results in a shift in nonverbalized, habitual, taken for granted frames of reference towards a perspective that is more open, reflective, and capable of change” ^61^  “The intended results of the interventions. Things that don’t exist now, but need to exist in order for the logical causal pathway not to be broken and the impact achieved. Example: Changes in knowledge, attitudes and skills of health workers to enable them to successfully deliver the intervention.” ^62^  “graduates must also know how to use the knowledge they have accumulated, for otherwise they may be little more than “idiot savants”. They must develop, among other things, the skill of acquiring information from a variety of human and laboratory sources, to analyze and interpret these data, and finally to translate such findings into a rational diagnostic or management plan. It is this quality of being functionally adequate, or of having sufficient knowledge, judgment, skill, or strength for a particular duty that Webster defines as competence” (p. s63) ^63^  Good quality research is likely to have improved outcomes for patients and is therefore important for quality patient care. ^34^  Awareness of the concept of using research in policy, knowledge and skills in the actions to engage with and apply research in policy/program agenda setting, development, implementation or evaluation ^65^ | 1, 2, 4, 6, 7, 14, 25, 26, 27, 29, 30 | 11 | Knowledge utilization |
| Changes in policy | Structural changes to facilitate the uptake of the innovation | The policy proposal makes it onto the governmental agenda. ^23^  To make difference, information mush influence the actions of policymakers, who should fight for the adoption of study’s recommendations. Adoption – whether the information influence policy outcomes (from Knott and Wildavsky, 1980, cited in 6).  Utilization of the information must change the way the policymakers sees the world, the understanding of the probabilities or magnitudes of impact (from Knott and Wildavsky, 1980, cited in 6)  The use of the dissemination object, can be categorized into conceptual use, making the effort to use, procedural use, structural use ^51^.  Possible outcomes of research utilization may include how the evidence is used in decision making (resource allocation/reallocation; maintain, discontinue or initiate programs/services; and staff training), and the decision-making process itself. ^19^  Ensure that policies, structures, and monies are aligned to support ^53^  Product innovation, leading to the substitution of old goods and services or to the satisfaction of new needs, is the main mechanism behind structural changes in economy (20)  We can hypothesize that incentive and reward systems have an indirect and positive effect on use of information. That is, the more organizations have incentives for using information, the more decision makers will use it by seeking information beyond their own agencies ^60^  Use of research to help identify which issues or problems are priorities and should be addressed by policy/program development ^65^ | 5, 6, 7, 14, 16, 20, 24b, 30 | 8 | Changes in policy, economics |
| Adoption | The individual or organization engages in a number of activities that will lead to the research evidence being integrated into clinical practice and/or policy decisions | Implementation takes place when an individual puts an innovation into use. Adoption is the decision to make full use of an innovation, rejection is a decision not to adopt an innovation ^16^  Use of evidence-based programs, incentive to use the programs ^45^  The individual or organization may choose not to adopt the innovation (but may revisit the decision at a later date as additional evidence is disseminated, or if information from the confirmation stage suggests adoption is warranted) or may choose to adopt the innovation in whole, in part or in some modified way. ^19^  If the decision to adopt the innovation is taken, then the individual or organization engages in a number of activities that will lead to the research evidence being integrated into clinical practice and/or policy decisions. This represents the implementation stage. ^19^  adoption is not an all-or-nothing process ^19^  “(..) elements [that] can accurately predict what a graduate does when functioning independently in a clinical practice. This action component of professional behavior is clearly the most difficult to measure accurately and reliably” (p. S63) ^63^ | 1, 9, 14, 27 | 4 | Adoption |
| Fidelity | To what extent were the various intervention components delivered as intended (in the protocol) | At the individual level, measures of participant follow-through or "adherence" to regimens are necessary for interpreting study outcomes. (13a)  Implementation (setting/agent level) - To what extent were the various intervention components delivered as intended (in the protocol), especially when conducted by different (nonresearch) staff members in applied settings? ^33^ | 1 | 1 | Fidelity, adherence |
| Confirmation | When an individual or an organization seeks reinforcement of an innovation-decision that has already been made | “Confirmation occurs when an individual seeks reinforcement of an innovation-decision that has already been made, but he or she may reverse this previous decision if exposed to conflicting messages about the innovation” ^16^  the individual or organization seeks to evaluate the consequences of adopting or rejecting the innovation (…) ^19^  the individual or organization seeks reinforcement for the decision made (…) Observability refers to the evaluation of the consequences of adopting the innovation ^19^ | 1, 14 | 2 | Confirmation |
| Accountability | Establishing clear responsibilities and expectations for stakeholders | In the eyes of the beholder (e.g., government, agencies, research community) balancing research, funding, expertise and time to conduct research ^22^  Establish clear responsibilities and expectation for stakeholders ^53^  “Level at which you stop using indicators to measure whether the outcomes have been achieved and therefore stop accepting responsibility for achieving those outcomes. The ceiling of accountability is often drawn between the impact and the long term outcome” ^62^ | 4, 16, 26 | 3 | Accountability |
| Impact | When the uptake of the innovation have tangible benefits | A policy may be implemented but fail to have the desired effects. Utilization takes place when information that yields tangible benefits to the citizen (from Knott and Wildavsky, 1980, cited in 6)  “reinvention” by an adopting organization during the innovation ^44^  Fit the innovation within the larger narrative by highlighting the contributions of youth to organizational success. ^53^  “The real-world change you are trying to affect. The program may contribute towards achieving this impact, and not achieve it solely on its own” (26  Good quality research is likely to have improved outcomes for patients and is therefore important for quality patient care. ^34^ | 6. 8. 16, 26, 29 | 5 | Impact |
| Maintenance, long term outcome | The extent to which a program or policy becomes institutionalized or part of the routine organizational practices and policies | The extent to which a program or policy becomes institutionalized or part of the routine organizational practices and policies (Setting); The long-term effects of a program on outcomes after 6 or more months after the most recent intervention contact (Individual) ^18,33^  “The final outcome the program is able to change on its own. This will be the primary outcome of the evaluation.” ^62^  Better health systems and health outcomes ^65^ | 13a, 26, 30 | 3 | Maintenance |
| Cost | Cost of the dissemination process | Policy “proposals that fail to meet these criteria—technical feasibility, value acceptability within the policy community, tolerable cost, anticipated public acquiescence, and a reasonable chance for receptivity among elected decision-makers—are not likely to be considered as serious, viable proposals” (p. 131). ^23^  Types of evidence: evidence of need established a health behavior problem as a public health priority, and includes the size, severity and cost of the problem. Evidence of demand reference key constructs from marketing and diffusion of innovation ^45^  Possible outcomes of research utilization may include how the evidence is used in decision-making (resource allocation/reallocation; maintain, discontinue or initiate programs/services; and staff training), and the decision-making process itself. ^19^  In terms of transaction cost economics, it means that the higher the costs supported by researchers to adapt their products, the lower the costs supported by the practitioners and professionals and, as a consequence, the higher the use of social science knowledge ^48^  Several examples can now be cited showing where improving quality of care reduces costs of care per patient ^40^ | 5, 9, 14, 24a, 31 | 5 | Cost |

Table A6. Frequency of Process Constructs Across Frameworks.

| Frameworks | Knowledge Inquiry | Knowledge Synthesis | Communication | Interaction | Persuading | Activation | Research transfer | Frequency of constructs |
| --- | --- | --- | --- | --- | --- | --- | --- | --- |
| 1 |  | 1 | 1 |  |  |  | 1 | 3 |
| 2 |  |  |  |  |  |  |  | 0 |
| 3 |  |  |  |  |  |  |  | 0 |
| 4 |  |  |  | 1 | 1 |  | 1 | 3 |
| 5 |  |  |  |  |  | 1 |  | 1 |
| 6 |  |  |  | 1 |  |  |  | 1 |
| 7 |  |  |  |  |  |  |  | 0 |
| 8 |  |  | 1 |  |  |  |  | 1 |
| 9 |  |  |  |  |  |  |  | 0 |
| 10 |  |  |  |  |  |  |  | 0 |
| 11 |  |  |  | 1 |  |  |  | 1 |
| 12 | 1 | 1 |  |  |  |  |  | 2 |
| 13a |  |  |  |  |  |  |  | 0 |
| 13b |  |  |  |  |  |  |  | 0 |
| 14 |  |  |  | 1 |  |  | 1 | 2 |
| 15 |  | 1 |  |  |  |  |  | 1 |
| 16 |  |  |  |  |  |  |  | 0 |
| 17 |  |  |  |  |  |  | 1 | 1 |
| 18 |  |  |  |  |  |  |  | 0 |
| 19 |  |  |  |  |  |  |  | 0 |
| 20 |  |  |  |  |  |  |  | 0 |
| 21 | 1 |  |  |  | 1 | 1 | 1 | 4 |
| 22 |  |  |  |  |  |  |  | 0 |
| 23 |  |  |  |  |  |  |  | 0 |
| 24a | 1 |  |  | 1 |  |  |  | 2 |
| 24b |  |  |  | 1 |  |  |  | 1 |
| 25 |  |  |  |  |  |  |  | 0 |
| 26 | 1 |  |  |  |  |  |  | 1 |
| 27 |  | 1 |  |  |  |  |  | 1 |
| 28 | 1 |  |  |  |  |  |  | 1 |
| 29 |  |  |  |  |  |  |  | 0 |
| 30 |  |  |  | 1 |  |  |  | 1 |
| 31 |  |  |  |  |  |  |  | 0 |
| 32 |  |  |  |  |  |  |  | 0 |
| 33 |  |  |  |  |  |  |  | 0 |
| 34 |  | 1 |  |  |  |  |  | 1 |
| 35 |  |  |  |  |  |  |  | 0 |
| 36 | 1 |  |  |  |  |  |  | 1 |
| 37 |  |  | 1 |  |  |  |  | 1 |
| Total frequency | 6 | 5 | 3 | 7 | 2 | 2 | 5 |  |

Table A7. Frequency of Determinant Constructs Across Frameworks.

| Frameworks | Source of knowledge | Medium of communication | Content of communication | Audience | Type of innovation | Complexity of the innovation | Timing of dissemination object | Urgency of the innovation | Triability of an innovation | Observability of the results | Salience of the innovation | Users’ perceived attitude towards the innovation | Compatibility of the innovation with the setting | Context | Interpersonal Networks | Opinion Leaders and Change Agents | Capacity | Total constructs |
| --- | --- | --- | --- | --- | --- | --- | --- | --- | --- | --- | --- | --- | --- | --- | --- | --- | --- | --- |
| 1 | 1 | 1 |  | 1 |  | 1 | 1 |  | 1 | 1 | 1 | 1 | 1 |  | 1 | 1 |  | 12 |
| 2 | 1 | 1 | 1 | 1 |  |  | 1 | 1 |  |  |  |  |  | 1 |  |  |  | 7 |
| 3 | 1 | 1 | 1 | 1 |  |  | 1 |  |  |  |  | 1 |  |  | 1 |  |  | 7 |
| 4 | 1 |  | 1 | 1 |  |  |  |  |  |  | 1 | 1 |  |  | 1 |  |  | 6 |
| 5 |  |  | 1 |  |  |  | 1 |  |  |  | 1 |  |  |  | 1 |  |  | 4 |
| 6 |  |  | 1 | 1 |  |  |  | 1 |  |  | 1 | 1 |  |  |  |  |  | 5 |
| 7 | 1 | 1 | 1 |  |  |  |  |  |  |  | 1 | 1 |  | 1 | 1 |  |  | 7 |
| 8 | 1 | 1 | 1 | 1 |  |  |  |  |  |  |  | 1 |  | 1 | 1 | 1 |  | 8 |
| 9 | 1 |  |  |  |  |  |  |  |  |  | 1 | 1 |  |  |  |  |  | 3 |
| 10 |  |  | 1 |  |  |  |  |  | 1 | 1 | 1 |  | 1 | 1 |  |  |  | 6 |
| 11 |  | 1 | 1 | 1 |  |  |  | 1 |  |  | 1 | 1 |  | 1 | 1 |  |  | 8 |
| 12 |  |  |  |  |  |  |  |  |  |  |  |  |  |  |  |  |  | 0 |
| 13a |  |  |  | 1 |  |  |  |  |  |  |  |  |  |  |  |  |  | 1 |
| 13b | 1 |  | 1 | 1 |  |  | 1 |  |  |  |  |  |  |  |  |  |  | 4 |
| 14 | 1 |  |  |  |  | 1 |  | 1 | 1 |  | 1 | 1 | 1 |  | 1 | 1 |  | 9 |
| 15 |  | 1 |  |  |  |  |  |  |  |  | 1 |  |  | 1 |  |  | 1 | 4 |
| 16 |  |  |  |  |  |  |  |  |  |  |  |  |  |  | 1 | 1 |  | 2 |
| 17 |  |  |  |  |  |  |  | 1 |  |  | 1 | 1 | 1 |  |  |  |  | 4 |
| 18 |  |  |  |  |  |  |  |  |  |  |  |  |  |  |  |  | 1 | 1 |
| 19 |  |  |  |  |  |  |  |  |  |  |  |  |  |  |  |  |  | 0 |
| 20 |  |  |  |  | 1 |  |  |  |  |  |  |  |  |  |  |  |  | 1 |
| 21 |  |  |  |  |  |  |  | 1 |  |  |  |  |  | 1 |  |  |  | 2 |
| 22 |  |  |  |  |  |  |  |  |  |  |  |  |  |  |  |  |  | 0 |
| 23 |  |  |  |  |  |  |  |  |  |  |  |  |  |  |  |  |  | 0 |
| 24a |  |  |  |  |  | 1 |  |  |  |  |  |  |  | 1 | 1 |  |  | 3 |
| 24b |  |  |  |  | 1 |  |  | 1 |  |  | 1 | 1 |  | 1 |  | 1 |  | 6 |
| 25 |  | 1 |  |  |  |  |  |  |  |  |  |  |  |  | 1 |  |  | 2 |
| 26 |  |  |  |  |  |  |  |  |  |  |  |  |  |  |  |  |  | 0 |
| 27 |  |  |  |  |  |  |  |  |  |  |  |  |  |  |  |  |  | 0 |
| 28 |  |  | 1 |  |  |  |  |  |  |  |  |  |  | 1 |  |  |  | 2 |
| 29 |  |  | 1 | 1 | 1 |  |  |  |  |  | 1 | 1 |  | 1 |  |  |  | 6 |
| 30 |  |  | 1 |  |  |  |  |  |  |  |  | 1 |  | 1 | 1 | 1 | 1 | 6 |
| 31 |  |  |  |  |  |  |  |  |  |  |  |  |  |  |  |  |  | 0 |
| 32 |  |  |  |  |  | 1 |  |  |  |  | 1 | 1 |  |  |  |  |  | 3 |
| 33 |  |  |  |  |  |  |  |  |  |  |  |  |  |  |  |  |  | 0 |
| 34 |  | 1 |  |  |  |  |  |  |  |  |  |  |  | 1 |  | 1 |  | 3 |
| 35 |  |  |  |  |  |  |  |  |  |  |  |  |  |  |  |  |  | 0 |
| 36 |  |  |  |  |  |  |  |  |  |  |  |  |  |  |  |  |  | 0 |
| 37 | 1 |  | 1 |  |  |  |  |  |  |  |  |  |  |  |  | 1 |  | 3 |
| Total frequency | 10 | 9 | 14 | 10 | 3 | 4 | 5 | 7 | 3 | 2 | 14 | 14 | 4 | 13 | 12 | 8 | 3 |  |

Table A8. Frequency of Strategy Constructs Across Frameworks.

| Frameworks | Identify the quality gap | Assess Dissemination Determinants | Assess     determinants of innovation uptake | Adapt the dissemination object to the context | Funding | Policy change | Monitoring and Evaluation | Sustain Knowledge Use | Increase audience’s skills | Total constructs |
| --- | --- | --- | --- | --- | --- | --- | --- | --- | --- | --- |
| 1 |  |  |  |  |  |  |  | 1 |  | 1 |
| 2 |  |  |  |  |  |  |  |  |  | 0 |
| 3 |  |  |  |  |  |  |  |  |  | 0 |
| 4 |  |  |  |  | 1 | 1 | 1 |  |  | 3 |
| 5 |  |  |  |  |  |  | 1 |  |  | 1 |
| 6 |  |  |  |  |  | 1 | 1 |  |  | 2 |
| 7 |  |  |  |  |  | 1 | 1 |  |  | 2 |
| 8 |  |  |  |  |  |  |  |  |  | 0 |
| 9 |  |  |  |  |  | 1 | 1 |  |  | 2 |
| 10 |  |  |  |  |  |  | 1 |  |  | 1 |
| 11 |  |  |  |  | 1 |  |  |  |  | 1 |
| 12 | 1 | 1 |  |  |  |  | 1 |  |  | 3 |
| 13a |  |  |  |  |  |  |  |  |  | 0 |
| 13b |  |  |  |  |  |  |  |  |  | 0 |
| 14 | 1 |  |  |  | 1 | 1 |  |  | 1 | 4 |
| 15 |  |  |  | 1 |  |  |  |  | 1 | 2 |
| 16 | 1 |  | 1 | 1 | 1 |  | 1 |  | 1 | 6 |
| 17 | 1 |  |  |  |  |  |  |  |  | 1 |
| 18 |  |  |  |  |  |  |  |  |  | 0 |
| 19 |  |  |  |  |  |  |  |  |  | 0 |
| 20 |  |  |  |  |  |  |  |  |  | 0 |
| 21 |  |  |  |  |  |  | 1 |  |  | 1 |
| 22 |  |  |  |  |  |  |  |  |  | 0 |
| 23 |  |  |  |  |  |  |  |  |  | 0 |
| 24a |  | 1 |  | 1 |  |  |  |  |  | 2 |
| 24b |  |  |  |  |  |  |  |  |  | 0 |
| 25 | 1 |  |  | 1 |  |  |  |  | 1 | 3 |
| 26 |  |  | 1 |  |  |  |  |  |  | 1 |
| 27 |  |  |  |  |  |  |  |  | 1 | 1 |
| 28 |  |  |  |  |  |  | 1 | 1 |  | 2 |
| 29 |  |  |  |  |  |  |  |  | 1 | 1 |
| 30 | 1 | 1 |  |  | 1 | 1 | 1 |  |  | 5 |
| 31 |  |  |  |  |  |  |  |  |  | 0 |
| 32 |  |  |  |  |  |  |  |  |  | 0 |
| 33 |  |  |  |  |  |  | 1 |  |  | 1 |
| 34 |  |  |  |  |  |  |  |  |  | 0 |
| 35 |  |  |  |  |  |  |  |  |  | 0 |
| 36 |  |  |  |  |  |  |  |  |  | 0 |
| 37 |  |  |  | 1 |  |  | 1 |  |  | 2 |
| Total Frequency | 6 | 3 | 2 | 5 | 5 | 6 | 13 | 2 | 6 |  |

Table A9. Frequency of Determinant Constructs Across Frameworks.

| Frameworks | Awareness | Reception | Persuasion | Emotion reactions | Decision | Knowledge gained | Knowledge utilization | Changes in policy | Adoption | Fidelity | Confirmation | Accountability | Impact | Maintenance, long term outcome | Cost | Total constructs |
| --- | --- | --- | --- | --- | --- | --- | --- | --- | --- | --- | --- | --- | --- | --- | --- | --- |
| 1 |  |  | 1 |  | 1 | 1 | 1 |  | 1 | 1 | 1 |  |  |  |  | 7 |
| 2 | 1 |  |  |  |  |  | 1 |  |  |  |  |  |  |  |  | 2 |
| 3 |  |  |  |  |  |  |  |  |  |  |  |  |  |  |  | 0 |
| 4 | 1 |  |  |  |  |  | 1 |  |  |  |  | 1 |  |  |  | 3 |
| 5 |  |  |  |  |  |  |  | 1 |  |  |  |  |  |  | 1 | 2 |
| 6 | 1 | 1 | 1 |  |  | 1 | 1 | 1 |  |  |  |  | 1 |  |  | 7 |
| 7 |  |  |  |  |  |  | 1 |  |  |  |  |  |  |  |  | 1 |
| 8 | 1 |  |  |  |  |  |  |  |  |  |  |  | 1 |  |  | 2 |
| 9 |  |  |  |  |  |  |  |  | 1 |  |  |  |  |  | 1 | 2 |
| 10 |  |  |  |  |  |  |  |  |  |  |  |  |  |  |  | 0 |
| 11 |  |  |  |  |  |  |  |  |  |  |  |  |  |  |  | 0 |
| 12 |  |  |  |  |  |  |  |  |  |  |  |  |  |  |  | 0 |
| 13a |  |  |  |  |  |  |  |  |  |  |  |  |  | 1 |  | 1 |
| 13b |  |  |  |  |  |  |  |  |  |  |  |  |  |  |  | 0 |
| 14 | 1 |  | 1 |  | 1 |  | 1 | 1 | 1 |  | 1 |  |  |  | 1 | 8 |
| 15 |  |  |  |  |  |  |  |  |  |  |  |  |  |  |  | 0 |
| 16 |  |  |  |  |  |  |  | 1 |  |  |  | 1 | 1 |  |  | 3 |
| 17 | 1 | 1 |  | 1 | 1 | 1 |  |  |  |  |  |  |  |  |  | 5 |
| 18 |  |  |  | 1 |  |  |  |  |  |  |  |  |  |  |  | 1 |
| 19 |  |  |  |  |  |  |  |  |  |  |  |  |  |  |  | 0 |
| 20 |  |  |  |  |  |  |  | 1 |  |  |  |  |  |  |  | 1 |
| 21 |  |  |  |  |  |  |  |  |  |  |  |  |  |  |  | 0 |
| 22 |  |  |  |  |  |  |  |  |  |  |  |  |  |  |  | 0 |
| 23 |  |  |  |  |  |  |  |  |  |  |  |  |  |  |  | 0 |
| 24a | 1 |  |  |  |  |  |  |  |  |  |  |  |  |  | 1 | 2 |
| 24b |  |  |  |  |  |  |  | 1 |  |  |  |  |  |  |  | 1 |
| 25 |  |  |  | 1 |  |  | 1 |  |  |  |  |  |  |  |  | 2 |
| 26 |  |  |  |  | 1 |  | 1 |  |  |  |  | 1 | 1 | 1 |  | 5 |
| 27 | 1 |  |  |  |  |  | 1 |  | 1 |  |  |  |  |  |  | 3 |
| 28 |  |  |  |  |  |  |  |  |  |  |  |  |  |  |  | 0 |
| 29 |  |  |  |  |  |  | 1 |  |  |  |  |  | 1 |  |  | 2 |
| 30 |  |  |  |  |  |  | 1 | 1 |  |  |  |  |  | 1 |  | 3 |
| 31 |  |  |  |  |  |  |  |  |  |  |  |  |  |  |  | 0 |
| 32 |  |  |  |  | 1 |  |  |  |  |  |  |  |  |  |  | 1 |
| 33 |  |  |  |  |  |  |  |  |  |  |  |  |  |  |  |  |
| Total Frequency | 8 | 2 | 3 | 3 | 5 | 3 | 11 | 7 | 4 | 1 | 2 | 3 | 5 | 3 | 4 |  |

**Preferred Reporting Items for Systematic reviews and Meta-Analyses extension for Scoping Reviews (PRISMA-ScR) Checklist**

| **SECTION** | **ITEM** | **PRISMA-ScR CHECKLIST ITEM** | **REPORTED ON PAGE #** |
| --- | --- | --- | --- |
| **TITLE** | | | |
| Title | 1 | Identify the report as a scoping review. | 1 |
| **ABSTRACT** | | | |
| Structured summary | 2 | Provide a structured summary that includes (as applicable): background, objectives, eligibility criteria, sources of evidence, charting methods, results, and conclusions that relate to the review questions and objectives. | 2 |
| **INTRODUCTION** | | | |
| Rationale | 3 | Describe the rationale for the review in the context of what is already known. Explain why the review questions/objectives lend themselves to a scoping review approach. | 4-5 |
| Objectives | 4 | Provide an explicit statement of the questions and objectives being addressed with reference to their key elements (e.g., population or participants, concepts, and context) or other relevant key elements used to conceptualize the review questions and/or objectives. | 5 |
| **METHODS** | | | |
| Protocol and registration | 5 | Indicate whether a review protocol exists; state if and where it can be accessed (e.g., a Web address); and if available, provide registration information, including the registration number. | N/A  We have not published a protocol paper for this review. |
| Eligibility criteria | 6 | Specify characteristics of the sources of evidence used as eligibility criteria (e.g., years considered, language, and publication status), and provide a rationale. | 5-6 |
| Information sources* | 7 | Describe all information sources in the search (e.g., databases with dates of coverage and contact with authors to identify additional sources), as well as the date the most recent search was executed. | 5-8 |
| Search | 8 | Present the full electronic search strategy for at least 1 database, including any limits used, such that it could be repeated. | 5-8 |
| Selection of sources of evidence† | 9 | State the process for selecting sources of evidence (i.e., screening and eligibility) included in the scoping review. | 5-8 |
| Data charting process‡ | 10 | Describe the methods of charting data from the included sources of evidence (e.g., calibrated forms or forms that have been tested by the team before their use, and whether data charting was done independently or in duplicate) and any processes for obtaining and confirming data from investigators. | 5-8 |
| Data items | 11 | List and define all variables for which data were sought and any assumptions and simplifications made. | 5-8 |
| Critical appraisal of individual sources of evidence§ | 12 | If done, provide a rationale for conducting a critical appraisal of included sources of evidence; describe the methods used and how this information was used in any data synthesis (if appropriate). | N/A |
| Synthesis of results | 13 | Describe the methods of handling and summarizing the data that were charted. | 8-11 |
| **RESULTS** | | | |
| Selection of sources of evidence | 14 | Give numbers of sources of evidence screened, assessed for eligibility, and included in the review, with reasons for exclusions at each stage, ideally using a flow diagram. | 8-11 |
| Characteristics of sources of evidence | 15 | For each source of evidence, present characteristics for which data were charted and provide the citations. | N/A |
| Critical appraisal within sources of evidence | 16 | If done, present data on critical appraisal of included sources of evidence (see item 12). | N/A |
| Results of individual sources of evidence | 17 | For each included source of evidence, present the relevant data that were charted that relate to the review questions and objectives. | 8-11 |
| Synthesis of results | 18 | Summarize and/or present the charting results as they relate to the review questions and objectives. | 8-11 |
| **DISCUSSION** | | | |
| Summary of evidence | 19 | Summarize the main results (including an overview of concepts, themes, and types of evidence available), link to the review questions and objectives, and consider the relevance to key groups. | 11-16 |
| Limitations | 20 | Discuss the limitations of the scoping review process. | 15 |
| Conclusions | 21 | Provide a general interpretation of the results with respect to the review questions and objectives, as well as potential implications and/or next steps. | 16 |
| **FUNDING** | | | |
| Funding | 22 | Describe sources of funding for the included sources of evidence, as well as sources of funding for the scoping review. Describe the role of the funders of the scoping review. | 16 |
